# Metabolomic Fingerprinting and UHPLC-ESI-HRMS/MS Profiling of Five Medicinal Plants Associated with Wound Care

**DOI:** 10.3390/plants15203164

**Published:** 2026-10-15

**Authors:** Iskra Davkova Stojanovski, Uroš Gašić, Danijela Mišić, Ana Trajkovska Matevska, Veronika Stoilkovska Gjorgievska, Filip Todorov, Ivana Cvetkovikj Karanfilova, Gjoshe Stefkov, Marija Karapandzova

**Affiliations:** 1 Faculty of Pharmacy, Institute of Pharmacognosy, Ss. Cyril and Methodius University in Skopje, Mother Theresa 47, 1000 Skopje, North Macedonia; ana.trajkovska@ff.ukim.edu.mk (A.T.M.); vsgjorgievska@ff.ukim.edu.mk (V.S.G.); todorov03f@gmail.com (F.T.); ivanacvetkovikj@ff.ukim.edu.mk (I.C.K.); gost@ff.ukim.edu.mk (G.S.); marija_karapandzova@ff.ukim.edu.mk (M.K.); 2 Institute for Biological Research “Siniša Stanković”, National Institute of Republic of Serbia, Department of Plant Physiology, University of Belgrade, 11108 Belgrade, Serbiadmisic@ibiss.bg.ac.rs (D.M.)

**Keywords:** LC-MS/MS, herbal drugs, phenolics, flavonoids, iridoids

## Abstract

Chronic wounds remain a challenging medical problem due to the involvement of multiple biological processes that can delay normal tissue repair. Medicinal plants may contain diverse bioactive compounds with potential relevance for wound management. However, detailed characterization of their chemical composition is essential for understanding their biological potential. The phytochemical composition of five medicinal plants, *Verbena officinalis*, *Plantago major*, *Achillea millefolium*, *Hypericum perforatum*, and *Calendula officinalis*, was investigated using ultra-high-performance liquid chromatography coupled with high-resolution electrospray ionization tandem mass spectrometry (UHPLC-ESI-HRMS/MS). A total of 162 metabolites were identified across the investigated extracts, representing diverse classes including flavonoids, phenolic acids, phenylethanoid glycosides, xanthones, naphthodianthrones, acylphloroglucinols and iridoid glycosides. A comparison with the available literature data and databases confirmed previously reported constituents and revealed 115 unique compounds that had not previously been reported for at least one of the five investigated plant species. The obtained results demonstrate the remarkable chemical diversity of the selected medicinal plants and provide a comprehensive basis for further investigation of their biological activities and potential use in multicomponent herbal mixtures for wound management.

## 1. Introduction

Chronic wounds remain a major clinical challenge due to their complex and multifactorial nature. Delayed healing is commonly associated with prolonged inflammation, microbial infection, impaired vascularization, and metabolic alterations that interfere with normal tissue repair. Frequently occurring in patients with underlying conditions such as diabetes, vascular disorders, and autoimmune diseases, chronic wounds require therapeutic approaches capable of addressing several interconnected biological processes [1,2,3]. Medicinal plants have gained increasing attention as complementary therapeutic options due to their diverse bioactive compounds, accessibility, favorable safety profile, and potential suitability for long-term use. Detailed phytochemical characterization of medicinal plants is an important step toward understanding the compounds responsible for their biological effects and supporting further pharmacological investigations. An integrated analysis of traditional use and contemporary scientific evidence highlighted *Verbena officinalis*, *Plantago major*, *Achillea millefolium*, *Hypericum perforatum* and *Calendula officinalis* as medicinal plants relevant to wound care, with previous studies reporting antioxidant, anti-inflammatory, antimicrobial, and regenerative activities for these species. This reported evidence, together with their traditional use in wound care, provided the rationale for selecting the five herbal drugs for comprehensive phytochemical characterization. Although their relevance to wound care is supported by traditional use and previously reported pharmacological evidence, detailed phytochemical characterization is needed to better define the chemical composition of these plants and provide a basis for subsequent investigations of their biological properties [4]. Comprehensive profiling can reveal the chemical diversity of the selected herbal drugs and identify major phytochemical classes and individual constituents that may warrant further biological investigation. Such characterization provides a detailed chemical framework for subsequent studies aimed at investigating the relationship between the phytochemical composition of these plants and their biological properties, including those relevant to wound care.

Given the chemical complexity of herbal extracts, analytical approaches capable of simultaneously detecting and characterizing a broad spectrum of structurally diverse metabolites are required. Therefore, ultra-high-performance liquid chromatography coupled with high-resolution electrospray ionization tandem mass spectrometry (UHPLC-ESI-HRMS/MS) was used to investigate the phytochemical composition of the selected medicinal plants. This approach enabled the characterization of secondary metabolites belonging to different phytochemical classes and provided a detailed overview of the chemical diversity of the investigated extracts, thereby establishing their molecular fingerprints and providing a phytochemical basis for further investigation of their biological properties.

## 2. Results

### 2.1. Identification of Components in Herbal Extracts

The analysis of herbal extracts using UHPLC-ESI-HRMS/MS led to the identification of 162 components. Among these, 56 were classified as flavonoid *O*-glycosides, 21 as flavonoid aglycones, 18 as hydroxycinnamic acid derivatives, 16 as hydroxybenzoic acid derivatives, and 10 as phenylethanoid glycosides. Additionally, 5 components were identified as xanthone aglycones, 4 as flavonoid *C*-glycosides, 3 as iridoid glycosides, 3 as xanthone glycosides, 2 as naphthodianthrones, and 17 as acylphloroglucinols, and 7 belonged to other classes of secondary metabolites. The identified components in herbal extracts are given in Table S1.

**Figure 1:**
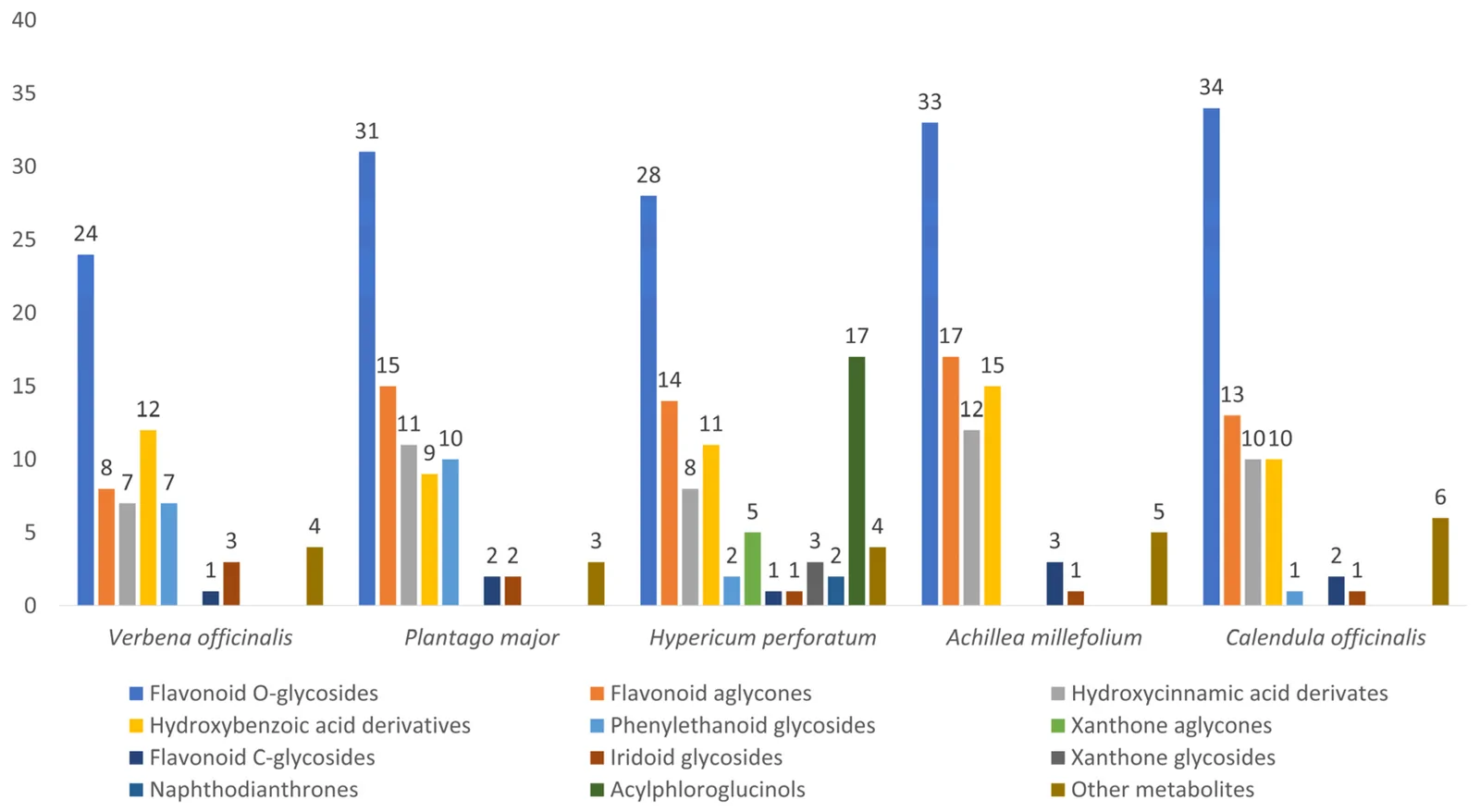
Representation of groups of secondary metabolites in plant extracts.

A total of 65 secondary metabolites were identified in an extract from *V. officinalis*, 83 in *P. major*, 96 in *H. perforatum*, 85 in *A. millefolium*, and 77 *in C. officinalis*. Phenolic compounds were the most commonly identified secondary metabolites, with flavonoid *O*-glycosides being the most represented group (Figure 1). Specifically, 24 flavonoid *O*-glycosides were identified in Vo, 31 in Pm, 28 in Hp, 33 in Am, and 34 in Co.

### 2.2. Chemometric Analysis of the LC-MS Data

#### 2.2.1. Hierarchical Cluster Analysis (HCA) of the Investigated Extracts

To gain deeper insights into the metabolic similarities and variations among the investigated plant species, a hierarchical cluster analysis (HCA) was performed on the log-transformed and autoscaled untargeted LC-MS dataset comprising 162 secondary metabolites. The resulting heatmap and two-way dendrogram (Figure S1) demonstrate a distinct grouping of both samples and metabolite features, reflecting characteristic species-specific phytochemical profiles.

As shown in the horizontal dendrogram, all injection triplicates of each species clustered closely together, indicating high analytical repeatability, while the grouping of samples into distinct branches reflected differences in their metabolic patterns (Figure S1). The species formed clear, well-separated main clusters: *Achillea* and *Calendula* samples grouped within closer proximity due to overlapping profiles of specific phenolic acids and flavonoid derivatives, whereas *Hypericum*, *Plantago*, and *Verbena* formed separate, highly divergent branches reflecting their unique specialized metabolite signatures.

The vertical dendrogram classified the 162 metabolites into distinct clusters based on their accumulation patterns across the investigated species (Figure S1). The bottom cluster of the heatmap is overwhelmingly dominated by specialized metabolites unique to *Hypericum*, featuring a massive accumulation block of characteristic prenylated phloroglucinols (such as hyperforin, adhyperforin, furohyperforin, hyperscabin variants, and hyperfirin), naphthodianthrones (hypericin, pseudohypericin), and specialized xanthone derivatives, alongside flavonoids like mangiferin, catechin, and myricetin. The upper and middle sections of the heatmap reveal robust accumulation blocks corresponding to flavonol and flavone glycosides, including diverse isorhamnetin, kaempferol, and quercetin hexosides and pentosyl-hexuronides, as well as chlorogenic and dicaffeoylquinic acids, which drive the close chemotaxonomic association between *Achillea* and *Calendula*. *Plantago* and *Verbena* exhibit distinct blocks characterized by iridoid glycosides and phenylpropanoid esters such as verbascoside, leucosceptoside A, and betonyoside B, which clearly segregate them from the flavonoid- and phloroglucinol-rich profiles of the other taxa.

#### 2.2.2. Principal Component Analysis (PCA) of the Investigated Extracts

To evaluate the overall data structure, natural grouping, and variance among the analyzed botanical samples, an unsupervised principal component analysis (PCA) was performed on the log-transformed and autoscaled LC-MS dataset comprising 162 secondary metabolites.

**Figure 2:**
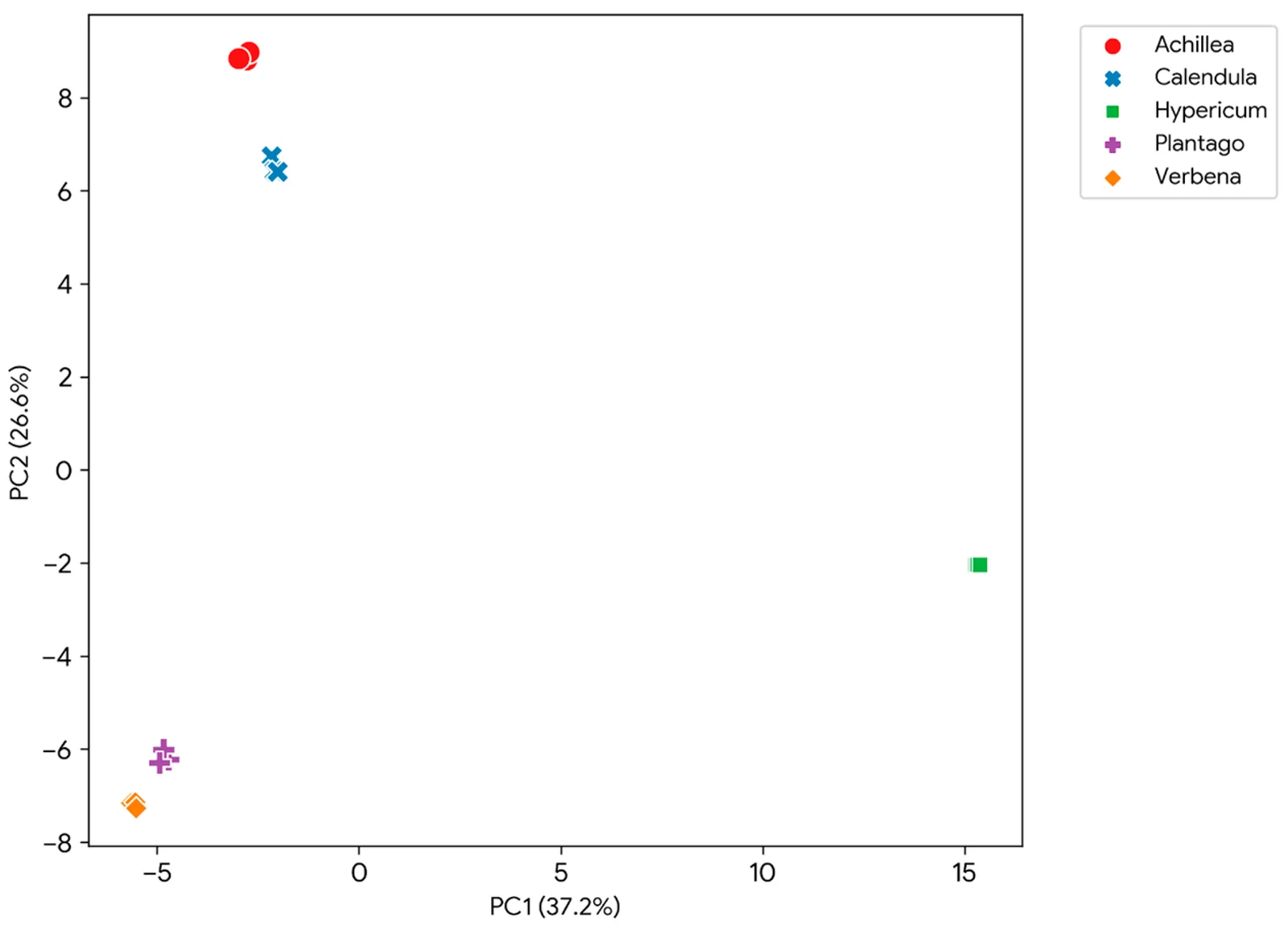
Principal component analysis (PCA) score plot derived from untargeted LC-MS profiles of the investigated plant species (*Achillea*, *Calendula*, *Hypericum*, *Plantago*, and *Verbena* analyzed in injection triplicates). The plot displays the spatial distribution of samples along the first two principal components (PC1 and PC2), accounting for 37.2% and 26.6% of the total variance, respectively.

As illustrated in the PCA score plot (Figure 2), the first two principal components explained a cumulative 63.8% of the total variance (37.2% for PC1 and 26.6% for PC2). The score plot reveals tight clustering of injection triplicates within each species, indicating high analytical repeatability. Furthermore, a clear separation among the species is evident: *Achillea* and *Calendula* samples group closely together due to overlapping phytochemical profiles rich in common flavonoids and phenolic acids. In contrast, *Plantago* and *Verbena* are clearly segregated along the principal axes, reflecting their distinct profile of phenylpropanoids and iridoid glycosides, while *Hypericum* forms an isolated cluster on the positive side of PC1, driven by its unique xanthone-dominated signature.

Due to the high density of features (162 metabolites), a loading plot with individual labels was omitted for visual clarity; instead, the variables driving the spatial distribution were evaluated through their respective loading coordinates. The separation along the first principal component (PC1) was primarily governed by the high positive and negative loadings distinguishing the unique metabolite classes (such as specialized xanthones in *Hypericum* versus phenylpropanoid derivatives and flavonoids in the other taxa). Meanwhile, the separation along the second principal component (PC2) was predominantly influenced by the specific accumulation patterns of flavonoids and phenolic acid derivatives characterizing *Achillea* and *Calendula*.

To further identify the specific variables responsible for the observed spatial separation, the corresponding loading values for all 162 metabolites along the principal components were evaluated (detailed in Figure S2 of the Supplementary Materials). The separation along PC1 (37.2% of total variance) was primarily driven by specialized metabolites characteristic of *Hypericum perforatum*, with prenylated phloroglucinols (such as hyperforin, hyperscabin C, and hyperwilsone F) and specialized xanthones (ugaxanthone, dihydroxy-methoxyxanthone variants) exhibiting the highest loading weights on the positive axis. Conversely, negative loadings along PC1 were associated with widespread flavonoid and phenolic derivatives found across the other taxa. Meanwhile, separation along PC2 (26.6% of total variance) was largely dictated by specific phenolic acid conjugates and flavone/flavonol glycosides, where dicaffeoylquinic acid 1, caffeoyl-feruloylquinic acid, and specific flavonoid glycosides (apigenin 6-*C*-hexoside-8-*C*-pentoside) contributed heavily on the positive side, whereas hemiphroside A and ferulic acid exerted strong negative loadings, distinguishing *Achillea* and *Calendula* from *Plantago* and *Verbena*.

## 3. Discussion

### 3.1. LC-MS Phytochemical Metabolite Profiling

UHPLC-ESI-HRMS/MS analysis revealed a chemically diverse metabolite profile across the investigated plant extracts, comprising predominantly phenolic constituents together with iridoid glycosides as a distinct class of terpenoid metabolites. The identified metabolites showed marked differences in their qualitative distribution among the investigated plant species (Figure 1).

**Figure 3:**
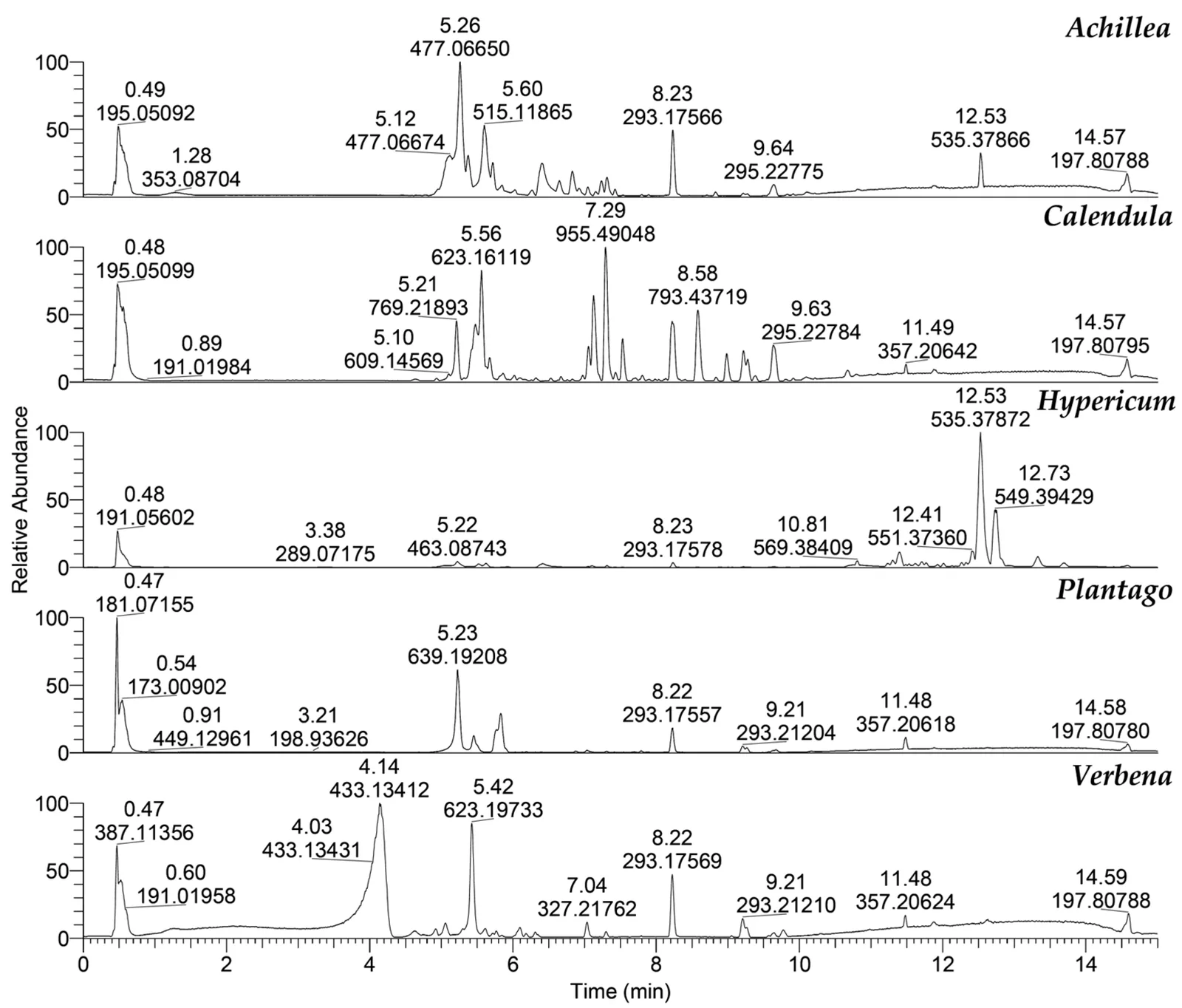
Representative base peak chromatograms of five investigated plants.

Flavonoid *O*-glycosides were the most abundant subclass across the analyzed extracts, with *Calendula officinalis* showing the highest number of the 34 identified compounds. Naphthodianthrones represented the least-represented class and were detected exclusively in *Hypericum perforatum*, with two identified compounds. Flavonoid aglycones constituted another major group across the analyzed extracts, except in *Verbena officinalis*, where hydroxybenzoic acid derivatives predominated. Acylphloroglucinols were also detected exclusively in *Hypericum perforatum*, where they represented a major compound group comprising 17 identified compounds. Hydroxycinnamic acid derivatives were widely detected, while phenylethanoid glycosides were mainly detected in *Plantago major* and *Verbena officinalis*, with 10 and 7 identified compounds, respectively. Base peak chromatograms of all five investigated plant extracts are depicted in Figure 3.

#### 3.1.1. *Verbena officinalis* Metabolite Profile

The phytochemical profile obtained for *Verbena officinalis* was consistent with the chemically diverse composition previously described for this species, with a total of 65 components identified in the analyzed extract. This number is higher than those reported in most previous studies (13–23 components) [5,6,7,8,9], but lower than the numbers reported by Stojković et al. (75 components) [10] and Xiao et al. (126 components) [11]. According to the available literature, hydroxybenzoic acid derivatives previously identified in Vo extracts include gallic acid [10,11], hydroxybenzoic acid, vanilloyl hexoside, dihydroxybenzoyl hexoside, and dihydroxybenzoyl pentoside [10], as well as 3,4-dihydroxybenzoic acid [12]. Previously reported hydroxycinnamic acid derivatives include ferulic acid [10,11], rosmarinic acid [5,10] and dicaffeoylquinic acid [5]. Additionally, previous phytochemical investigations of this extract have reported the presence of phenylethanoid glycosides, including verbascoside [6,7,8,10], 2′-acetyl-verbascoside [7] and leucosceptoside A [8], as well as iridoid glycosides such as verbenalin [7,8,10] and dihydroverbenalin [7,8]. Flavonoids and flavonoid aglycones previously reported in the available literature include apigenin 7-*O*-(2″-hexuronyl)-hexuronide, isorhamnetin 3-*O*-(6″-rhamnosyl)-glucoside, apigenin 7-*O*-hexuronide [6], quercetin 3-*O*-(6″-rhamnosyl)-glucoside, luteolin 7-*O*-hexuronide, luteolin 7-*O*-glucoside, isorhamnetin 3-*O*-hexuronide, luteolin 7-*O*-(2″-hexuronyl)-hexuronide [10], apigenin 7-*O*-hexuronide [6,10] and vitexin [13]. Reported flavonoid aglycones include luteolin [8,10], naringenin [10], chrysoeriol [11], quercetin [8,10], apigenin [8,10,11], kaempferol [8] and acacetin [7]. Other metabolites, such as quinic acid and benzoyl-malic acid, have also been detected in *V. officinalis* extracts [10]. All compounds previously reported for Vo were also detected in the extract analyzed in the present study, confirming the consistency of its phytochemical profile with earlier reports. However, several constituents described in the literature were not detected, including pedalitin and scutellarein [5,10], as well as hastatoside [5,9,10,11], aucubin [5,9] and isoverbascoside [5,9,10]. In addition, the present study revealed six hydroxybenzoic acid derivatives that were not previously reported in the reviewed literature, including hydroxybenzoyl hexoside 1, hydroxybenzoyl hexoside 2, syringoyl hexoside 1, dihydroxybenzoic acid–benzoyl hexoside, 4-hydroxybenzoic acid and galloyl pentoside. The hydroxybenzoic acid derivatives generally showed fragmentation characterized by loss of glycosidic residues, followed by formation and subsequent fragmentation of the corresponding phenolic acid-related ions. Hydroxybenzoyl hexosides 1 and 2 exhibited analogous fragmentation patterns, characterized by the neutral loss of a hexosyl moiety, yielding the hydroxybenzoic acid-related ion at *m*/*z* 137, which subsequently underwent decarboxylation to produce the respective MS^2^ base peaks at *m*/*z* 93.03457 and 93.03426. Similarly, syringoyl hexoside 1 showed the characteristic neutral loss of 162 Da, generating the syringic acid-related ion at *m*/*z* 197.04526. In the case of 4-hydroxybenzoic acid, decarboxylation through the loss of CO_2_ resulted in the MS^2^ base peak at *m*/*z* 93.03420. Finally, galloyl pentoside retained the deprotonated molecular ion as the base peak, while product ions at *m*/*z* 168.00720 and 125.02444 supported the presence of a galloyl-related structural moiety. Furthermore, the hydroxycinnamic acid profile comprised four derivatives that have not been previously described in the reviewed literature for this extract, including caffeoyl hexosyl-hexoside, caffeoyl hexoside, 3-*O*-caffeoylquinic acid and nepetoidin B. The hydroxycinnamic acid derivatives showed fragmentation patterns predominantly characterized by the loss of glycosidic or ester-linked residues, followed by formation of characteristic caffeic acid- and quinic acid-related product ions. Caffeoyl hexosyl-hexoside and caffeoyl hexoside exhibited closely related fragmentation patterns, generating the characteristic caffeic acid ion at *m*/*z* 179.03514 and 179.03532, respectively, following cleavage of the corresponding sugar residues. Subsequent fragmentation produced ions at approximately *m*/*z* 161 and 135, consistent with dehydration and decarboxylation of the caffeic acid-related ion, respectively. 3-*O*-Caffeoylquinic acid generated the predominant ion at *m*/*z* 191.05537, corresponding to deprotonated quinic acid following the loss of the caffeoyl residue, together with the caffeic acid-related ions at *m*/*z* 179.03435 and 135.04466, supporting its proposed annotation. Nepetoidin B produced the predominant ion at *m*/*z* 161.02463, together with the product ion at *m*/*z* 133.03006, consistent with the formation and subsequent fragmentation of a hydroxycinnamoyl-related structural unit and supporting the proposed annotation. The present study also expanded the phenylethanoid glycoside profile of the extract by revealing four constituents that were not documented in the reviewed literature, including oxoverbascoside, plantamajoside, betonyoside B and hemiphroside A. The phenylethanoid glycosides showed fragmentation characterized by the loss of sugar and hydroxycinnamoyl-related residues, accompanied by the formation of characteristic lower-mass product ions. Oxoverbascoside generated the prominent product ion at *m*/*z* 477.14011, corresponding to a neutral loss of approximately 160 Da from the precursor ion, together with the characteristic hydroxycinnamoyl-related ion at *m*/*z* 161.02438. Plantamajoside retained the deprotonated molecular ion as the base peak, while the prominent product ion at *m*/*z* 161.02374, together with additional lower-mass fragments, supported its proposed identification as a phenylethanoid glycoside. Betonyoside B similarly retained an intense precursor ion and generated a product ion at *m*/*z* 477.16077, together with ions at *m*/*z* 193.05052, 175.03998, 161.02429, and 160.01653. Hemiphroside A also retained the deprotonated molecular ion as the base peak, while product ions at *m*/*z* 175.03996 and 160.01654 further supported its proposed assignment. Deoxyloganic acid was the only iridoid glycoside not previously reported in the reviewed literature for this extract. Its MS^2^ spectrum was dominated by the product ion at *m*/*z* 197.08195, corresponding to a hexosyl residue. Subsequent fragmentation generated ions at *m*/*z* 153.09209, 135.08165, and 109.06591, further supporting the proposed identification of deoxyloganic acid. Moreover, apigenin 8-*C*-glucoside was identified as an additional flavonoid, *C*-glycoside, that was not previously reported in the reviewed literature for Vo. In contrast to *O*-glycosides, its MS^2^ fragmentation was characterized by cross-ring cleavage of the *C*-linked sugar residue rather than complete cleavage of the glycosidic bond. The product ions at *m*/*z* 311.05499 and 283.06009 were consistent with this characteristic fragmentation behavior and supported its proposed identification as an apigenin *C*-glycoside. A total of sixteen flavonoid *O*-glycosides that were not previously reported in the reviewed literature for Vo were identified, including myricetin 3-*O*-hexoside, taxifolin 3-*O*-rhamnoside, isosakuranetin 7-*O*-(6″-rhamnosyl)-hexoside, quercetin 3-*O*-hexuronide-7-*O*-hexoside, quercetin 3-*O*-(2″,6″-dirhamnosyl)-hexoside, kaempferol 3-*O*-pentoside-7-*O*-hexoside, quercetin 3-*O*-galactoside, kaempferol 3-*O*-(6″-rhamnosyl)-hexoside, isorhamnetin 3-*O*-(2″-pentosyl)-hexuronide, isorhamnetin 3-*O*-(2″-acetyl-6″-rhamnosyl)-hexoside, apigenin 7-*O*-(2″-pentosyl)-hexoside, chrysoeriol 7-*O*-(6″-rhamnosyl)-hexoside, quercetin 7-*O*-hexoside, isorhamnetin 3-*O*-(6″-malonyl)-hexoside, apigenin 7-*O*-(6″-caffeoyl)-hexoside and 3′,8″-biapigenin. The flavonoid *O*-glycosides generally showed fragmentation characterized by cleavage of their sugar residues and formation of characteristic aglycone-related product ions. Myricetin 3-*O*-hexoside generated the ion at *m*/*z* 316.02252 following a neutral loss of 162 Da, whereas taxifolin 3-*O*-rhamnoside produced characteristic taxifolin-related fragments, including the prominent ion at *m*/*z* 151.00374. Isosakuranetin 7-*O*-(6″-rhamnosyl)-hexoside generated the aglycone-related ion at *m*/*z* 285.07590 following the overall loss of the rhamnosyl-hexosyl residues, with subsequent fragmentation producing the ion at *m*/*z* 270.05282. Quercetin derivatives were characterized by product ions at approximately *m*/*z* 301 and 300. Quercetin 3-*O*-hexuronide-7-*O*-hexoside showed sequential losses consistent with hexuronyl and hexosyl residues, generating the intermediate ion at *m*/*z* 463.09552 and the quercetin-related ion at *m*/*z* 301.03561. Quercetin 3-*O*-(2″,6″-dirhamnosyl)-hexoside similarly generated ions at *m*/*z* 301.03470 and 300.02759 following the loss of its glycosidic substituents. Quercetin 3-*O*- galactoside and quercetin 7-*O*-hexoside both generated characteristic ions at approximately *m*/*z* 301 following neutral losses of 162 Da. Kaempferol and isorhamnetin derivatives displayed analogous glycosidic cleavage patterns. Kaempferol 3-*O*-pentoside-7-*O*-hexoside generated the intermediate ion at *m*/*z* 417.10254 following loss of a hexosyl residue, with subsequent fragmentation producing the kaempferol-related ion at *m*/*z* 284.03357. Kaempferol 3-*O*-(6″-rhamnosyl)-hexoside similarly produced ions at *m*/*z* 285.04047 and 284.03271 following the overall loss of 308 Da. The isorhamnetin derivatives were characterized by diagnostic ions at approximately *m*/*z* 315, 314, and 300 following cleavage of their respective substituents. Notably, isorhamnetin 3-*O*-(6″-malonyl)-hexoside generated the prominent ion at *m*/*z* 519.11438 through decarboxylation of the malonyl group, together with characteristic isorhamnetin-related product ions. The apigenin and chrysoeriol *O*-glycosides likewise generated characteristic aglycone-related ions following cleavage of their substituents. Apigenin 7-*O*-(2″-pentosyl)-hexoside and apigenin 7-*O*-(6″-caffeoyl)-hexoside produced the apigenin-related ions at *m*/*z* 269.04544 and 269.04605, respectively, while the additional ion at *m*/*z* 161.02501 in the latter supported the presence of a caffeoyl-related structural unit. Chrysoeriol 7-*O*-(6″-rhamnosyl)-hexoside generated the characteristic chrysoeriol-related ion at *m*/*z* 299.05606 following the overall loss of the rhamnosyl-hexosyl residues. The MS^2^ spectrum of biflavonoid 3′,8″-biapigenin showed product ions at *m*/*z* 385.07108, 267.02975, and 151.00362, consistent with fragmentation of the biflavonoid skeleton and supporting its proposed identification. Among the flavonoid aglycones, 6-hydroxykaempferol 3,6-dimethyl ether was not previously reported in the reviewed literature for Vo. The flavonoid aglycones generally exhibited fragmentation patterns characteristic of their respective flavonoid skeletons and substitution patterns. In particular, 6-hydroxykaempferol 3,6-dimethyl ether generated a prominent product ion at *m*/*z* 314.04117, corresponding to the loss of a methyl radical from a methoxy substituent and supporting the proposed identification of the methylated flavonoid. Finally, comparison with the reviewed literature indicated two additional metabolites with no previous reports for this extract, including tuberonic acid hexoside and phlorizin. Tuberonic acid hexoside generated the product ion at *m*/*z* 225.07605, consistent with cleavage of a hexosyl residue and formation of the tuberonic acid-related ion. Phlorizin showed an analogous neutral loss of 162 Da, generating the base peak at *m*/*z* 273.07578, corresponding to the deprotonated phloretin aglycone.

#### 3.1.2. *Plantago major* Metabolite Profile

A total of 83 components were identified in the extract of *Plantago major*, demonstrating its chemically diverse phytochemical profile. According to the available literature on the chemical characterization of this species, several phenolic acids and related compounds have been previously reported, including gallic acid [14], 3,4-dihydroxybenzoic acid, 4-hydroxybenzoic acid [15], 3-*O*-caffeoylquinic acid [16], rosmarinic acid [17], ferulic acid, *p*-coumaric acid [16] and quinic acid [15]. Additionally, several phenylethanoid glycosides have been documented, including verbascoside and plantamajoside [16,18], leucoseptoside [18] and helicoside [19]. The flavonoid profile reported for this extract is particularly diverse and includes the aglycones taxifolin [20], luteolin [15,20], quercetin [14,17], apigenin [15,17], isorhamnetin [14], naringenin [17] and hispidulin [16], together with a broad range of their glycosylated derivatives. These include hispidulin 7-*O*-hexuronide, luteolin 7-*O*-glucoside [13], luteolin 7-*O*-hexuronide [21], tricin 7-*O*-hexoside, quercetin 3-*O*-hexuronide, isorhamnetin 3-*O*-(6″-rhamnosyl)-glucoside, myricetin 3-*O*-hexoside [13], 3′,8″-biapigenin and isoquercetin, as well as vitexin [15] and apigenin 6-*C*-hexoside-8-*C*-hexoside [13]. The literature contains conflicting data regarding the relative abundance of plantamajoside and verbascoside, with some sources indicating the predominance of plantamajoside, while others report an equal distribution of both compounds. Structurally, plantamajoside (C_29_H_36_O_16_) differs from verbascoside (acteoside, C_29_H_36_O_15_) by the presence of glucose instead of rhamnose [22]. The phytochemical profile obtained for Pm was consistent with the chemically diverse composition previously described for this species, while further expanding the range of metabolites detected in the investigated extract. Additionally, five hydroxybenzoic acid derivatives not previously reported in the reviewed literature for broadleaf plantain were identified, including syringoyl hexoside 1, hydroxybenzoyl hexoside 1, dihydroxybenzoyl hexoside, dihydroxybenzoyl pentoside and vanilloyl hexoside. Dihydroxybenzoyl hexoside and dihydroxybenzoyl pentoside showed characteristic neutral losses of 162 and 132 Da, respectively, generating the dihydroxybenzoic acid-related ion at *m*/*z* 153.01935, which underwent further decarboxylation to *m*/*z* 109.02957/109.02965, followed by formation of the base peak at *m*/*z* 108.02169/108.02165. Vanilloyl hexoside exhibited the characteristic loss of a hexosyl moiety, resulting in the vanillic acid-related ion at *m*/*z* 167.03493, followed by demethylation to *m*/*z* 152.01152 and subsequent decarboxylation, producing the base peak at *m*/*z* 108.02166. The hydroxycinnamic acid profile of this extract was further expanded by the detection of seven compounds absent from previous reports, including caffeoyl hexosyl-hexoside, caffeoyl hexoside, feruloyl hexoside, dicaffeoylquinic acid 1, *p*-coumaroyl hexoside, *p*-coumaric acid–*p*-coumaroyl hexoside and nepetoidin B. Feruloyl hexoside generated the base peak at *m*/*z* 193.05095 following a neutral loss of a hexosyl residue and formation of the deprotonated ferulic acid-related ion. Dicaffeoylquinic acid 1 produced the prominent ion at *m*/*z* 353.08575, consistent with the loss of one caffeoyl-related residue, followed by formation of the quinic acid-related ion at *m*/*z* 191.05533. The *p*-coumaric acid derivatives showed characteristic product ions at approximately *m*/*z* 163 and 119. *p*-Coumaroyl hexoside generated the ion at *m*/*z* 163.03941 following cleavage of the hexosyl residue, with subsequent decarboxylation producing the base peak at *m*/*z* 119.04979. Similarly, *p*-coumaric acid–*p*-coumaroyl hexoside generated product ions at *m*/*z* 163.04036 and 119.05038, supporting the presence of *p*-coumaroyl-related structural units. Moreover, six phenylethanoid glycosides have not been previously reported in this species, including oxoverbascoside, betonyoside B, 2′-acetyl-verbascoside, hemiphroside A, lancetoside and hydroxy-scroside E. 2′-Acetyl-verbascoside similarly retained the deprotonated molecular ion as the base peak and generated the product ion at *m*/*z* 487.14694, corresponding to a neutral loss of approximately 178 Da. The additional ions at *m*/*z* 179.03493, 161.02434, and 135.04504 were consistent with fragmentation of a hydroxycinnamoyl-related structural unit. Lancetoside was characterized by the base peak at *m*/*z* 161.02379, accompanied by product ions at *m*/*z* 135.04443 and 133.02953, supporting fragmentation of a hydroxycinnamoyl-related structural unit. Hydroxy-scroside E retained the precursor ion as the base peak and generated an ion at *m*/*z* 651.20056, consistent with dehydration, together with lower-mass product ions at *m*/*z* 193.05128, 175.04068, 160.01680, and 151.03992, supporting its proposed identification. Comparison with previous reports further revealed two iridoid glycosides—dihydroverbenalin and deoxyloganic acid—which had not been previously documented for this extract. Dihydroverbenalin generated the dominant product ion at *m*/*z* 227.09171, consistent with loss of the glycosidic portion and formation of an iridoid-related fragment. Additional ions at *m*/*z* 195.06635, 125.02441, and 101.02412 reflected further fragmentation of the iridoid-related structure, supporting its proposed identification. Flavonoid *O*-glycosides represented a particularly diverse group of constituents with twenty-two compounds that were not previously documented in the reviewed literature for Pm. These included apigenin 7-*O*-(2″-hexuronyl)-hexuronide, taxifolin 3-*O*-rhamnoside, kaempferol 3-*O*-rhamnoside-7-*O*-rhamnoside, patuletin 3-*O*-hexoside, quercetin 3-*O*-(6″-malonyl)-hexoside, quercetin 3-*O*-pentoside, isorhamnetin 3-*O*-hexuronide, isorhamnetin 3-*O*-(2″-pentosyl)-hexuronide, isorhamnetin 3-*O*-(2″-acetyl-6″-rhamnosyl)-hexoside, apigenin 7-*O*-(2″-pentosyl)-hexoside, apigenin 7-*O*-hexuronide, quercetin 7-*O*-hexoside, chrysoeriol 7-*O*-hexoside, kaempferol 3-*O*-(6″-malonyl)-hexoside, quercetin 3-*O*-(2″-acetyl)-rhamnoside, isorhamnetin 3-*O*-(6″-malonyl)-hexoside, isorhamnetin 3-*O*-(6″-acetyl)-hexoside, tricin 7-*O*-hexoside, apigenin 7-*O*-(6″-acetyl)-hexoside, luteolin 7-*O*-(6″-hexuronyl)-hexoside, kaempferol 3-*O*-(6″-acetyl)-hexoside and apigenin 7-*O*-(6″-caffeoyl)-hexoside. Among the apigenin derivatives not previously discussed, apigenin 7-*O*-(2″-hexuronyl)-hexuronide generated the base peak at *m*/*z* 351.05603, together with the characteristic apigenin-related ion at *m*/*z* 269.04559, consistent with loss of the two hexuronyl residues. Apigenin 7-*O*-hexuronide produced the base peak at *m*/*z* 269.04523 following a neutral loss of 176 Da, whereas apigenin 7-*O*-(6″-acetyl)-hexoside generated ions at *m*/*z* 269.04669 and 268.03741 following cleavage of the acetylated hexosyl substituent. The additionally detected quercetin derivatives showed characteristic formation of quercetin-related ions at approximately *m*/*z* 301 and 300. Quercetin 3-*O*-(6″-malonyl)-hexoside generated the base peak at *m*/*z* 505.09848, together with ions at *m*/*z* 301.03497 and 300.02747, supporting the loss of the malonylated hexosyl substituent. Quercetin 3-*O*-pentoside produced ions at *m*/*z* 301.03546 and 300.02759 following a neutral loss of 132 Da, whereas quercetin 3-*O*-hexuronide generated the base peak at *m*/*z* 301.03525 following loss of a hexuronyl residue. Quercetin 3-*O*-(2″-acetyl)-rhamnoside produced the base peak at *m*/*z* 284.03238, accompanied by ions at *m*/*z* 285.04208, 255.03090, and 227.03516, supporting cleavage of the acetylated rhamnosyl substituent and subsequent fragmentation of the quercetin-related structure. Among the kaempferol derivatives, kaempferol 3-*O*-rhamnoside-7-*O*-rhamnoside generated intermediate ions at *m*/*z* 431.09942 and 430.08853, consistent with the loss of one rhamnosyl residue, followed by kaempferol-related ions at *m*/*z* 285.04065 and 283.02484. Kaempferol 3-*O*-(6″-malonyl)-hexoside produced the base peak at *m*/*z* 489.10098, consistent with decarboxylation of the malonyl substituent, followed by formation of ions at *m*/*z* 285.03961 and 284.03119. Kaempferol 3-*O*-(6″-acetyl)-hexoside generated characteristic product ions at *m*/*z* 301.03510 and 300.02759, together with lower-mass ions at *m*/*z* 271.02481, 255.02992, and 243.02997. Patuletin 3-*O*-hexoside generated the patuletin-related ion at *m*/*z* 331.04510 following a neutral loss of 162 Da, together with the ion at *m*/*z* 330.03809 and subsequent product ions at *m*/*z* 316.02274 and 315.01482. Among the isorhamnetin derivatives not previously discussed, isorhamnetin 3-*O*-hexuronide generated the base peak at *m*/*z* 315.04974 following loss of a hexuronyl residue, while isorhamnetin 3-*O*-(6″-rhamnosyl)-glucoside produced the characteristic isorhamnetin-related ions at *m*/*z* 315.05096, 314.04321, and 300.02756. Isorhamnetin 3-*O*-(6″-acetyl)-hexoside similarly generated ions at *m*/*z* 315.05109, 314.04333, and 300.02750 following cleavage of the acetylated hexosyl substituent. Finally, chrysoeriol 7-*O*-hexoside generated the ion at *m*/*z* 299.05643 following loss of a hexosyl residue, together with the prominent ion at *m*/*z* 284.03229, consistent with subsequent fragmentation involving the methoxy substituent. Tricin 7-*O*-hexoside produced product ions at *m*/*z* 313.03497, 285.03894, and 271.02484, supporting fragmentation of the methoxylated flavonoid structure. Luteolin 7-*O*-(6″-hexuronyl)-hexoside generated the base peak at *m*/*z* 285.04044, corresponding to the luteolin-related ion following cleavage of its glycosidic substituents and supporting its proposed identification. A comparison with the available literature distinguished eight more flavonoid aglycones as previously undocumented constituents of *P. major*, including chrysoeriol, kaempferol, chrysin, hesperetin, pectolinarigenin, acacetin, pinocembrin and 6-hydroxykaempferol 3,6-dimethyl ether. Chrysoeriol showed a characteristic loss of the methoxy substituent, generating the MS^2^ base peak at *m*/*z* 284.03156. Kaempferol retained the deprotonated molecular ion as the MS^2^ base peak and produced lower-mass ions at *m*/*z* 211.04059, 185.06065, 151.00446, 143.05022, and 117.03484, consistent with cleavage of the flavonoid *C*-ring and subsequent fragmentation of the aglycone skeleton. Chrysin generated product ions at *m*/*z* 145.02927, 143.04973, 119.04978, and 107.01316, likewise reflecting fragmentation of the flavonoid core. Hesperetin generated the product ion at *m*/*z* 286.04660, corresponding to a loss of approximately 15 Da associated with its methoxy substituent. Pectolinarigenin showed analogous behavior, producing the ion at *m*/*z* 298.04788 through a loss of approximately 15 Da, followed by formation of the ion at *m*/*z* 283.02600. Similarly, acacetin generated the characteristic product ion at *m*/*z* 268.03699, also corresponding to cleavage of the methoxy substituent. Pinocembrin produced product ions at *m*/*z* 213.05446, 171.04485, 151.00342, 145.06549, and 107.01347, consistent with cleavage of the flavanone *C*-ring and subsequent fragmentation of the flavonoid structure. Finally, the comprehensive phytochemical profiling revealed two additional other metabolites—tuberonic acid hexoside and aesculin—that had not been previously reported in the reviewed literature for broadleaf plantain. Aesculin produced the base peak at *m*/*z* 177.01859 following a cleavage of the hexosyl residue and formation of the deprotonated aesculetin-related ion. Additional product ions at *m*/*z* 133.02899 and 105.03421 were consistent with further fragmentation of the coumarin structure, supporting its proposed identification.

#### 3.1.3. *Hypericum perforatum* Metabolite Profile

The phytochemical analysis of *Hypericum perforatum* resulted in the identification of 96 compounds, considerably expanding the chemical profile reported in previous LC-MS-based studies, which identified 36 [23] and 42 compounds [24]. The identified constituents belonged to several major phytochemical classes, including phenolic acids and their derivatives, flavonoids and their glycosides, as well as other secondary metabolites. Among the phenolic acid derivatives previously reported in Hp were caffeoyl hexoside [24], 3-*O*-caffeoylquinic acid [25,26], 3-*O*-*p*-coumaroylquinic acid [24,26], *p*-coumaric acid [23], ferulic acid [23,27,28], dicaffeoylquinic acid 1 [24,26], rosmarinic acid [26], syringoyl hexoside 1, vanilloyl hexoside, dihydroxybenzoyl hexoside [24], gallic acid [23,24,28], 3,4-dihydroxybenzoic acid [23,24], and 4-hydroxybenzoic acid [23]. In addition, quinic acid [23,26,27] and phlorizin [29] have also been previously documented. Flavonoids represented another prominent group of previously reported constituents. Among the flavonoid aglycones, kaempferol [23,24,25], naringenin [24,27], acacetin, apigenin [23], isorhamnetin [13], quercetin [23,24,28,30,31], luteolin [23], myricetin [24] and catechin [23,24,26] have been previously described, together with the biflavonoid 3′,8″-biapigenin [24,30,31]. A considerable diversity of glycosylated flavonoids has also been documented, including quercetin 3-*O*-rhamnoside [24,26,27,32,33], isorhamnetin 3-*O*-(6″-rhamnosyl)-glucoside [23], quercetin 3-*O*-pentoside, quercetin 3-*O*-(6″-acetyl)-hexoside [24], kaempferol 3-*O*-(6″-rhamnosyl)-hexoside [23], taxifolin 3-*O*-rhamnoside [13,30], quercetin 3-*O*-hexuronide [30], quercetin 7-*O*-hexoside [34], isorhamnetin 3-*O*-glucoside [26], apigenin 7-*O*-glucoside [13,35], procyanidin dimer B type [24], quercetin 3-*O*-galactoside (hyperoside) [23,25,26,31] and myricetin 3-*O*-hexoside [24]. Moreover, characteristic compounds identified in Hp extracts included protopseudohypericin [24,30,32], hypericin [28,29,30,32], hyperforin [25,28,29,30,31,32], adhyperfirin [24,25,30,32], adhyperforin [25,28,29,30,31,32], furohyperforin [36], furohyperforin A, furoadhyperforin [37], hyperforatone I, hyperforatone J [38] and 32-*epi*-hyperforatin E [39]. The presence of all previously reported phytochemical components in the analyzed extract further supports their relevance as characteristic secondary metabolites of this species and confirms the consistency of the obtained phytochemical profile with previous LC-MS-based investigations. Furthermore, the phytochemical analysis revealed five hydroxybenzoic acid derivatives that were not previously reported in the reviewed literature on St. John’s wort: galloyl pentoside, dihydroxybenzoyl pentosyl-hexoside, hydroxybenzoyl hexoside 1, dihydroxybenzoyl pentoside and dihydroxybenzoic acid–dihydroxybenzoyl hexoside. Dihydroxybenzoyl pentosyl-hexoside retained the precursor ion as the MS^2^ base peak and generated product ions at *m*/*z* 152.01152 and 108.02167, consistent with fragmentation of a dihydroxybenzoyl-related structural unit. Dihydroxybenzoic acid–dihydroxybenzoyl hexoside similarly generated characteristic product ions at *m*/*z* 153.01936/152.01146 and 109.02947/108.02172, consistent with fragmentation of dihydroxybenzoic acid-related structural units and supporting its proposed identification. Among the hydroxycinnamic acid derivatives and flavonoid *C*-glycosides identified in this study, feruloyl hexoside and luteolin 8-*C*-pentoside were the only representatives of their respective classes that were not previously reported in the reviewed literature for this extract. The MS^2^ spectrum of this *C*-glycoside showed characteristic product ions at *m*/*z* 357.06128 and 327.05124, corresponding to losses consistent with cross-ring fragmentation of the *C*-linked pentosyl residue. Additionally, two phenylethanoid glycosides, plantamajoside and hemiphroside A, and one iridoid glycoside, deoxyloganic acid, were identified as constituents that were not previously documented in the reviewed literature for Hp. Furthermore, thirteen flavonoid *O*-glycosides were identified as previously unreported constituents, including quercetin 3,7-di-*O*-hexoside, quercetin 3-*O*-hexuronide-7-*O*-hexoside, quercetin 3-*O*-pentoside-7-*O*-hexoside, kaempferol 3-*O*-pentoside-7-*O*-hexoside, luteolin 7-*O*-hexuronide, quercetin 3-*O*-(6″-malonyl)-hexoside, chrysoeriol 7-*O*-(6″-rhamnosyl)-hexoside, kaempferol 3-*O*-(6″-malonyl)-hexoside, quercetin 3-*O*-(2″-acetyl)-rhamnoside, hispidulin 7-*O*-hexuronide, tricin 7-*O*-hexoside, quercetin 7-*O*-pentoside and kaempferol 3-*O*-(6″-acetyl)-hexoside. Among the derivatives not discussed above, quercetin 3,7-di-*O*-hexoside generated the base peak at *m*/*z* 463.08853 following a loss of one hexosyl residue, together with product ions at *m*/*z* 301.03433 and 300.02805, supporting subsequent loss of the second hexosyl residue and formation of quercetin-related ions. Quercetin 3-*O*-pentoside-7-*O*-hexoside similarly generated product ions at *m*/*z* 463.08615 and 433.07587, corresponding to the loss of pentosyl and hexosyl residues, respectively, followed by formation of the quercetin-related ions at *m*/*z* 301.03403 and 300.02631. Quercetin 7-*O*-pentoside produced the characteristic quercetin-related ions at *m*/*z* 301.03448 and 300.02725, consistent with cleavage of the pentosyl residue. Luteolin 7-*O*-hexuronide generated the base peak at *m*/*z* 285.04047 following a loss of the hexuronyl residue and formation of the deprotonated luteolin-related ion. Hispidulin 7-*O*-hexuronide generated the ion at *m*/*z* 299.05615 following a neutral loss of 176 Da, consistent with cleavage of the hexuronyl residue, while the base peak at *m*/*z* 284.03268 was consistent with subsequent fragmentation involving the methoxy substituent. The present study reveals additional five flavonoid aglycones that were not previously documented in the reviewed literature, including taxifolin, patuletin, hispidulin, chrysoeriol and pinocembrin. Taxifolin underwent initial dehydration, generating the product ion at *m*/*z* 285.03922, followed by *C*-ring cleavage and further fragmentation, yielding ions at *m*/*z* 217.04985, 177.01877, 175.03958, and 151.00314, with the diagnostic ion at *m*/*z* 125.02398 representing the MS^2^ base peak. Patuletin generated the product ion at *m*/*z* 316.02261, corresponding to a loss of its methoxy substituent. Hispidulin similarly generated the base peak at *m*/*z* 284.03159, supporting fragmentation involving eliminating the methoxy substituent. A distinct feature of the *H. perforatum* metabolite profile was the detection of seven xanthone derivatives not previously documented in the reviewed literature, comprising two xanthone glycosides and five aglycones. The glycosides included trihydroxyxanthone *C*-hexoside and trihydroxyxanthone *O*-hexoside. Their MS^2^ fragmentation reflected the different modes of sugar attachment. Trihydroxyxanthone *C*-hexoside generated the base peak at *m*/*z* 285.04102, consistent with cross-ring fragmentation of the *C*-linked hexosyl residue. In contrast, trihydroxyxanthone *O*-hexoside generated the base peak at *m*/*z* 243.03003 following the loss of the *O*-linked hexosyl residue and formation of the deprotonated xanthone aglycone. The five xanthone aglycones comprised trihydroxy-methoxyxanthone, dihydroxy-methoxyxanthone 1, dihydroxy-dimethoxyxanthone, dihydroxy-methoxyxanthone 2 and ugaxanthone. The two dihydroxy-methoxyxanthone isomers generated base peaks at *m*/*z* 242.02127 and 242.02098, respectively, corresponding to losses of approximately 15 Da from their precursor ions. Dihydroxy-dimethoxyxanthone showed analogous behavior, generating the base peak at *m*/*z* 272.03262 through a loss of approximately 15 Da. These transitions were consistent with fragmentation involving the methoxy substituents, while additional lower-mass product ions reflected further fragmentation of the xanthone skeleton and supported the proposed assignments. Another characteristic feature of the Hp metabolite profile was the detection of seven polycyclic polyprenylated acylphloroglucinol (PPAP) derivatives, including norhyperpalum E, hyperwilsone F, hookerione B, hyperpatulone M, hyperuralone F, hyperscabin F and hyperscabin C. Although these compounds have previously been reported from other *Hypericum* species, including *H. patulum*, *H. wilsonii*, *H. hookerianum*, *H. uralum* and *H. scabrum*, they have not been previously documented in *H. perforatum* in the reviewed literature [23,24,25,26,27,28,29,30,31,32,33,34,35,36,37,38,39,40,41,42,43,44,45,46]. Their detection therefore represents an additional characteristic feature of the phytochemical profile obtained in the present study and extends the occurrence of these structurally diverse PPAP derivatives within the genus *Hypericum*. Their MS^2^ spectra showed characteristic fragmentation patterns involving successive cleavage and rearrangement of the polyprenylated acylphloroglucinol skeleton. In particular, the formation of product ions corresponding to losses of approximately 68.06 Da in hyperpatulone M and hyperscabin F was consistent with the elimination of C_5_H_8_ units derived from isoprenyl moieties. The remaining PPAP derivatives generated characteristic series of higher- and lower-mass product ions, reflecting further fragmentation of the prenylated substituents and oxygenated polycyclic core. These fragmentation patterns, together with the accurate precursor masses, supported the proposed identification of the respective PPAP derivatives. Finally, the phytochemical profile was further complemented by two additional metabolites not previously documented in the reviewed literature for St. John’s wort: tuberonic acid hexoside and 5,7-dihydroxy-2-(2-methylpropyl)-4H-1-benzopyran-4-one. 5,7-Dihydroxy-2-(2-methylpropyl)-4H-1-benzopyran-4-one generated the base peak at *m*/*z* 189.05612 following a neutral loss of approximately 44 Da, consistent with CO_2_ loss and supporting its proposed identification.

#### 3.1.4. *Achillea millefolium* Metabolite Profile

Previous LC–MS-based phytochemical studies of *Achillea millefolium* have reported varying numbers of identified constituents, including 34 and 48 compounds [47,48], respectively, whereas a total of 85 compounds were identified in the extract analyzed in the present study. The existing literature consistently highlights several compounds belonging to different phytochemical classes as recurrently reported constituents of Yarrow extract. Among the hydroxybenzoic acid derivatives, previously reported compounds include vanilloyl hexoside, dihydroxybenzoyl hexoside, dihydroxybenzoyl pentoside, hydroxybenzoic acid [49], 3,4-dihydroxybenzoic acid [13,50] and ellagic acid [51]. Regarding hydroxycinnamic acid derivatives, the literature reports 3-*O*-caffeoylquinic acid [47,48,50,52,53], caffeic acid [50,53,54,55], *p*-coumaric acid [56,57], 5-*O*-feruloylquinic acid [48,49], dicaffeoylquinic acid [47,49,51,56], rosmarinic acid [53,54], sinapic acid [58] and caffeoyl hexoside [57]. Among flavonoid *C*-glycosides, vitexin [49,53] and apigenin 6-*C*-hexoside-8-*C*-pentoside [49,57] have previously been reported in Am. Flavonoid *O*-glycosides represent a particularly diverse group of previously reported constituents, including quercetin 3-*O*-(6″-acetyl)-hexoside, apigenin 7-*O*-(2″-pentosyl)-hexoside, apigenin 7-*O*-(6″-acetyl)-hexoside, isorhamnetin 3-*O*-(6″-acetyl)-hexoside [57], isorhamnetin 3-*O*-(6″-rhamnosyl)-hexoside, quercetin 3-*O*-hexuronide, quercetin 3-*O*-pentoside, quercetin 3-*O*-(2″-pentosyl)-hexuronide, chrysoeriol 7-*O*-hexoside [49], quercetin 3-*O*-pentoside-7-*O*-hexoside [56], quercetin 3-*O*-(6″-rhamnosyl)-glucoside [47,48,50,51,52,55,56], quercetin 3-*O*-hexoside [47,50,51,54,56], luteolin 7-*O*-hexuronide [47,50,53], luteolin 7-*O*-glucoside [47,48,50,51,53,55,56], kaempferol 3-*O*-(6″-rhamnosyl)-hexoside [47,58], quercetin 3-*O*-(6″-malonyl)-hexoside [54], quercetin 3-*O*-rhamnoside [47,54], apigenin 7-*O*-hexuronide [48,54,56] and chrysoeriol 7-*O*-(6″-rhamnosyl)-hexoside [48]. Among flavonoid aglycones, luteolin [47,48,51,53,55,56,58,59], quercetin [47,53,54], apigenin [47,48,51,53,54,55,58], centaureidin [53,58], naringenin [60,61], chrysoeriol [49,57], kaempferol [57], eriodictyol [49] and taxifolin [57,60] have also been previously documented. Moreover, quinic acid [49,51] and fraxidin [48] were also reported. The analysis of the extract used in our study confirmed the presence of all previously reported compounds, while also revealing a broader spectrum of secondary metabolites. Among these, nine hydroxybenzoic acid derivatives that were not previously documented in the reviewed literature for *A. millefolium* were identified, including galloyl pentoside, dihydroxybenzoyl pentosyl-hexoside, syringoyl hexoside 1, hydroxybenzoyl hexoside 1, gallic acid, hydroxybenzoyl hexoside 2, gallic acid–hydroxybenzoyl hexoside, dihydroxybenzoic acid–benzoyl hexoside and dihydroxybenzoic acid–dihydroxybenzoyl hexoside. Among the derivatives not discussed above, gallic acid generated the MS^2^ base peak at *m*/*z* 125.02396, corresponding to the neutral loss of CO_2_ from the deprotonated molecule. Gallic acid–hydroxybenzoyl hexoside retained the precursor ion as the MS^2^ base peak and yielded product ions at *m*/*z* 331.06699 and 313.05594, along with characteristic fragments at *m*/*z* 168.00600, 137.02415, and 125.02401. The ions at *m*/*z* 168 and 125 were attributed to the gallic acid-related structural unit, with the latter arising through decarboxylation, whereas the ion at *m*/*z* 137 was consistent with a hydroxybenzoic acid-related moiety. The hydroxycinnamic acid profile of this extract was further characterized by three constituents absent from previous reports in the reviewed literature: caffeoyl hexosyl-hexoside, caffeoyl-feruloylquinic acid and nepetoidin B. Among the derivatives not discussed above, caffeoyl-feruloylquinic acid generated the base peak at *m*/*z* 367.10269 following a neutral loss of approximately 162 Da, while product ions at *m*/*z* 353.08664, 193.05052, 191.05606, and 173.04538 were consistent with fragmentation involving caffeoyl-, feruloyl-, and quinic acid-related structural units, supporting its proposed identification. Deoxyloganic acid and luteolin 8-*C*-pentoside were the additional constituents not previously documented among the iridoid glycosides and flavonoid *C*-glycosides. A considerably broader diversity was observed among flavonoid *O*-glycosides, with fourteen compounds not previously documented in the reviewed literature for Am. These included quercetin 3-*O*-hexuronide-7-*O*-hexoside, kaempferol 3-*O*-pentoside-7-*O*-hexoside, taxifolin 3-*O*-rhamnoside, patuletin 3-*O*-hexuronide, patuletin 3-*O*-hexoside, isorhamnetin 3-*O*-hexuronide, isorhamnetin 3-*O*-(2″-pentosyl)-hexuronide, quercetin 7-*O*-hexoside, kaempferol 3-*O*-(6″-malonyl)-hexoside, quercetin 3-*O*-(2″-acetyl)-rhamnoside, isorhamnetin 3-*O*-(6″-malonyl)-hexoside, tricin 7-*O*-hexoside, eriodictyol-3′,8″-quercetin and 3′,8″-biapigenin. Among the derivatives not discussed above, patuletin 3-*O*-hexuronide generated the base peak at *m*/*z* 331.04581 following a neutral loss of 176 Da, consistent with the loss of the hexuronyl residue and formation of the patuletin-related ion. Eriodictyol-3′,8″-quercetin generated the base peak at *m*/*z* 285.03949, accompanied by product ions at *m*/*z* 301.03525, 151.00374, and 133.02946, consistent with fragmentation of the constituent flavonoid-related structural units and supporting its proposed assignment. The aglycone fraction comprised five flavonoids not previously documented in the reviewed literature for this extract, including patuletin, axillarin, hesperetin, 6-hydroxykaempferol 3,6-dimethyl ether and pinocembrin. Axillarin generated the base peak at *m*/*z* 330.03790 following a loss of approximately 15 Da from the precursor ion, consistent with fragmentation involving a methoxy substituent and supporting its proposed identification. Finally, the phytochemical analysis revealed three additional metabolites—aesculin, tuberonic acid hexoside, and phlorizin—that have not been previously described for *A. millefolium* in the reviewed literature, further broadening the currently known chemical composition of this species.

#### 3.1.5. *Calendula officinalis* Metabolite Profile

The phytochemical profiling of *Calendula officinalis* extract resulted in the identification of a total of 77 components, revealing a chemically diverse composition. Previous LC–MS-based investigations of Marigold have differed considerably in their analytical scope. While several studies employed targeted approaches for the analysis of predefined phenolic compounds, Al-Huqail et al. tentatively identified 20 constituents in a methanolic flower extract [62]. More recently, Ossipov et al., using UPLC-PDA-Q Exactive Orbitrap-HRMS/MS, characterized 58 major metabolites in ligulate and tubular flowers, comprising 15 phenolic compounds, 7 triterpenoid glycosides, and 36 relatively polar lipids [63]. Among the hydroxybenzoic acids, 3,4-dihydroxybenzoic acid [64] and 4-hydroxybenzoic acid [62,65] have been previously reported in Co. The hydroxycinnamic acid profile described in the literature comprises 3-*O*-caffeoylquinic acid [63,66,67], caffeic acid [62,64,66,67,68,69], *p*-coumaric acid [65,66,68], dicaffeoylquinic acid 1 and 2 [62,67], sinapic acid and rosmarinic acid [65,66]. Regarding flavonoids, vitexin [66] has been previously documented as a flavonoid *C*-glycoside, while a considerably broader range of flavonoid *O*-glycosides has been reported. These include quercetin 3-*O*-rutinosyl-rhamnoside, isorhamnetin 3-*O*-rutinosyl-rhamnoside [63], quercetin 3-*O*-(6″-rhamnosyl)-glucoside [62,64,68,69,70], quercetin 3-*O*-hexoside [63,64,68,70,71], kaempferol 3-*O*-rutinoside [63,64], quercetin 3-*O*-(6″-malonyl)-hexoside [71], isorhamnetin 3-*O*-rutinoside [62,63], quercetin 3-*O*-glucuronide [62,64], isorhamnetin 3-*O*-glucoside [70,71], kaempferol 3-*O*-glucoside [64], isorhamnetin 3-*O*-(6″-malonyl)-hexoside [71], apigenin 7-*O*-glucoside [64], patuletin 3-*O*-hexoside, quercetin 7-*O*-hexoside, chrysoeriol 7-*O*-hexoside [72], quercetin 3-*O*-(6″-acetyl)-hexoside, and isorhamnetin 3-*O*-(6″-acetyl)-hexoside [67]. In addition to these glycosylated derivatives, the flavonoid aglycones luteolin [64], quercetin [62,64,66,68], apigenin, kaempferol [66] and isorhamnetin [68] have also been documented, as well as quinic acid [64]. Phytochemical profiling of the extract used in the present study confirmed the presence of all previously reported compounds, while also revealing eight additional hydroxybenzoic acid derivatives: galloyl pentoside, syringoyl hexoside 1, hydroxybenzoyl hexoside 1, vanilloyl hexoside, dihydroxybenzoyl hexoside, dihydroxybenzoyl pentoside, dihydroxybenzoic acid–dihydroxybenzoyl hexoside and hydroxybenzoic acid. Characteristic decarboxylation of hydroxybenzoic acid through the neutral loss of CO_2_ generated the MS^2^ base peak at *m*/*z* 93.03461. The hydroxycinnamic acid fraction further expanded the metabolite profile of Co, with three compounds not previously documented in the reviewed literature: caffeoyl hexoside, caffeoyl-feruloylquinic acid and dicaffeoylquinic acid 3. Among these, dicaffeoylquinic acid 3, whose fragmentation has not been discussed above, generated the characteristic base peak at *m*/*z* 353.08740, corresponding to the loss of one caffeoyl-related unit from the precursor ion. Additional product ions at *m*/*z* 191.05617, 179.03502, 173.04579, and 135.04521 were consistent with quinic acid- and caffeic acid-related structural units and supported the proposed identification. The analysis also revealed previously undocumented constituents across several phytochemical classes. Among the phenylethanoid glycosides, plantamajoside emerged as an additional component, while deoxyloganic acid broadened the iridoid glycoside composition of *C. officinalis*. Within the flavonoid *C*-glycoside subclass, apigenin 6-*C*-hexoside-8-*C*-pentoside was identified as an additional constituent that was not previously documented in the reviewed literature for this extract. Its MS^2^ spectrum showed characteristic cross-ring cleavages of the *C*-linked sugar residues, resulting in product ions at *m*/*z* 383.07651 and 353.06696, corresponding to cumulative neutral losses of 180 and 210 Da from the precursor ion, respectively, supporting its assignment as a di-*C*-glycosylated apigenin derivative. The greatest expansion, however, was observed among the flavonoid *O*-glycosides, with seventeen additional compounds identified beyond those previously described for Marigold including quercetin 3-*O*-pentoside-7-*O*-hexoside, isorhamnetin 3-*O*-(6″-hexosyl)-hexoside-7-*O*-hexoside, isorhamnetin 3-*O*-(6″-hexosyl)-hexoside, isorhamnetin 3-*O*-(2″-pentosyl-6″-rhamnosyl)-hexoside, kaempferol 3-*O*-pentoside-7-*O*-hexoside, luteolin 7-*O*-hexuronide, quercetin 3-*O*-pentoside, isorhamnetin 3-*O*-(2″-pentosyl)-hexuronide, quercetin 3-*O*-rhamnoside, isorhamnetin 3-*O*-(2″-acetyl-6″-rhamnosyl)-hexoside, isorhamnetin 3-*O*-pentoside-7-*O*-hexoside, kaempferol 3-*O*-(6″-malonyl)-hexoside, isorhamnetin 3-*O*-(2″-caffeoyl-6″-pentosyl)-hexoside, quercetin 3-*O*-(2″-acetyl)-rhamnoside, hispidulin 7-*O*-hexuronide, tricin 7-*O*-hexoside and 3′,8″-biapigenin. Among the derivatives whose fragmentation has not been discussed above, the highly glycosylated isorhamnetin derivatives predominantly retained their precursor ions as the MS^2^ base peaks while generating characteristic isorhamnetin-related product ions. Isorhamnetin 3-*O*-(6″-hexosyl)-hexoside-7-*O*-hexoside generated an ion at *m*/*z* 623.16107, together with ions at *m*/*z* 315.05219, 314.04373, and 299.01935, whereas isorhamnetin 3-*O*-(6″-hexosyl)-hexoside produced ions at *m*/*z* 315.05319, 314.04642, and 299.02206. Isorhamnetin 3-*O*-(2″-pentosyl-6″-rhamnosyl)-hexoside similarly generated characteristic ions at *m*/*z* 315.05121, 314.04297, 300.02542, and 299.01987. Isorhamnetin 3-*O*-pentoside-7-*O*-hexoside produced ions at *m*/*z* 314.04370 and 299.02026, further supporting the presence of the isorhamnetin skeleton. Quercetin 3-*O*-rhamnoside (quercitrin) retained the precursor ion as the MS^2^ base peak at *m*/*z* 447.09311 and generated characteristic quercetin-related ions at *m*/*z* 301.03534 and 300.02726, consistent with cleavage of the rhamnosyl residue, together with lower-mass product ions at *m*/*z* 178.99861 and 151.00347. Isorhamnetin 3-*O*-(2″-caffeoyl-6″-pentosyl)-hexoside also retained the precursor ion as the MS^2^ base peak at *m*/*z* 771.17902 and generated a product ion at *m*/*z* 609.14215, corresponding to a neutral loss of approximately 162 Da, together with isorhamnetin-related ions at *m*/*z* 315.05029 and 314.04474. These product ions supported the proposed identification of the respective flavonoid *O*-glycosides. The aglycone fraction comprised eight flavonoids that were not previously documented in the reviewed literature for Co: taxifolin, eriodictyol, axillarin, naringenin, chrysoeriol, 6-hydroxykaempferol 3,6-dimethyl ether, centaureidin and acacetin. Among the aglycones whose fragmentation has not been discussed above, eriodictyol underwent characteristic cleavage of the flavanone *C*-ring, yielding diagnostic ions at *m*/*z* 151.00314, 135.04468, and 107.01360, with *m*/*z* 135.04468 representing the MS^2^ base peak. Similarly, naringenin showed characteristic *C*-ring fragmentation, generating the diagnostic product ions at *m*/*z* 151.00400 and 119.04977, with the former as the MS^2^ base peak. Centaureidin exhibited successive losses of approximately 15 Da from the precursor ion, producing ions at *m*/*z* 344.05304, 329.03064, and 314.00699, consistent with sequential loss of methyl radicals from its methoxy substituents, followed by further fragmentation to *m*/*z* 301.03543, 286.01193, and 258.01660. Beyond these phytochemical classes, five other constituents not previously documented in the reviewed literature were identified, further enriching the metabolite profile of *Calendula officinalis*: aesculin, tuberonic acid hexoside, fraxidin, benzoyl-malic acid and 5,7-dihydroxy-2-(2-methylpropyl)-4H-1-benzopyran-4-one. Among these, fraxidin generated the MS^2^ base peak at *m*/*z* 206.02202, corresponding to a loss of approximately 15 Da from the precursor ion and consistent with fragmentation involving a methoxy substituent. Additional product ions at *m*/*z* 190.99850, 163.00352, 135.00885, and 107.01387 reflected further fragmentation of the coumarin skeleton and supported the proposed identification. Benzoyl-malic acid showed a characteristic cleavage of the ester linkage involving the malic acid moiety, generating the benzoyl-related ion at *m*/*z* 121.02963 as the MS^2^ base peak.

A comparison with the available LC-MS-based literature confirmed the presence of many characteristic metabolites previously described for these five species, while also revealing an additional 34 compounds for Vo, 52 for Pm, 44 for Hp, 36 for Am and 44 for Co. These findings provide a broader understanding of the phytochemical composition of the selected medicinal plants and further demonstrate the complexity of their secondary metabolite profiles. The presence of multiple metabolite classes highlights the chemical diversity of these extracts and provides a basis for further investigation of their biological properties. Several compounds detected in the investigated species have previously been reported to exhibit antioxidant, antimicrobial and anti-inflammatory activities in independent studies [73,74,75,76,77]. Their detection provides a basis for investigating the biological properties of the extracts and their potential relevance to wound care. Experimental studies should therefore evaluate the activities of the individual extracts and determine whether combinations of chemically distinct extracts produce complementary or synergistic effects.

## 4. Materials and Methods

### 4.1. Plant Materials

The plant materials used in this study were dried aerial parts (leaves, stems, and flowers) of *Verbena officinalis* L., Verbenaceae (Vo) with voucher specimen number FFUKIMVo1/22, *Hypericum perforatum* L., Hypericaceae (Hp) with FFUKIMHp1/22 and *Achillea millefolium* L., Asteraceae (Am) FFUKIMAm1/22, and *Plantago major* L., Plantaginaceae (Pm) FFUKIMPm1/22 dried leaves and *Calendula officinalis* L., Asteraceae (Co)—FFUKIMCo1/22 dried flowers. Hp, Am and Co were supplied by Alkaloid AD Skopje, North Macedonia, while Vo and Pm were purchased from a herbal pharmacy in Skopje, North Macedonia. The dried herbal materials were purchased in bulk and supplied by weight, in quantities of approximately 1 kg per plant material.

### 4.2. Extractive Procedures

Extraction of the plant raw materials was done from 0.5 g of dry and ground plant material with 10 mL of 50% EtOH (*v*/*v*), prepared by dilution of 96% ethanol supplied by Alkaloid AD, Skopje, North Macedonia, sonicated in an ultrasonic bath (Selecta 0476619, 720 W, 50/60 Hz; Barcelona, Spain) at a temperature of 25 °C for 15 min, and then filtered through filter paper and supplemented with 50% EtOH (*v*/*v*), up to 10 mL in a volumetric flask.

### 4.3. Chemical Characterization of Extracts Using UHPLC-ESI-HRMS/MS

Identification of the components present in the plant extracts was performed using a liquid chromatography method with ultra-high performance in conjunction with mass spectrometry with high resolution for electrospray ionization (UHPLC-ESI-HRMS/MS).

UHPLC-ESI-HRMS/MS analyses were performed using a Hypersil GOLD™ C18 column (50 × 2.1 mm, particle size 1.9 μm, Thermo Finnigan, San Jose, CA, USA), at a temperature of 40 °C, an injection volume of 5 μL and with a constant flow rate of 300 μL/min. The mobile phases used were: (A) 0.1% formic acid in water and (B) 0.1% formic acid in acetonitrile. The compounds of interest were eluted with ultrapure water +0.1% formic acid (A) and acetonitrile (MS grade) + 0.1% formic acid (B): 5% B in the first min; 5–95% B from 1 to 10 min; 95% B from 10 to 12 min; 5% B until 15 min.

The operating conditions for the Orbitrap Exploris 120 mass spectrometer (Thermo Finnigan, San Jose, CA, USA), equipped with an ESI source, operating in negative ionization mode were: a spray voltage of 4500 V, a capillary temperature of 325 °C and a RF tube lens of 50%. Nitrogen (30 arbitrary units—AU), auxiliary (10 AU) and purge gas (1 AU) were used for the envelope. MS scans were performed from 100 to 1500 *m*/*z* with the Orbitrap resolution set to 60,000 FWHM, while MS^2^ scans were performed with the Orbitrap resolution of 15,000 FWHM. The spectra were measured at several collision energies with the higher energy set to 15, 30 and 45 absolute units, with an isolation width of 1.5 *m*/*z*. The dynamic cut-off time was set to 10 s, with exclusion from a specific scan after two occurrences, and the intensity threshold was set to 1 × 105. Identification of phenolic compounds was made by comparing the retention times (Rt) and molecular (M-H)^-^ and fragment ions (MS^2^) of the analyzed compounds with the corresponding standard substances, for which the software Xcalibur ^®^ 2.20 version (Thermo Finnigan, San Jose, CA, USA) was used.

### 4.4. Chemometric Analysis

#### 4.4.1. Hierarchical Cluster Analysis (HCA)

To evaluate the hierarchical relationships and metabolic similarities among the investigated plant species (*Achillea*, *Calendula*, *Hypericum*, *Plantago*, and *Verbena* in injection triplicates), a Hierarchical Cluster Analysis (HCA) was performed on the log-transformed and autoscaled untargeted LC-MS dataset comprising 162 secondary metabolites. The distance matrix and sample/feature clustering were generated using Morpheus online software (https://software.broadinstitute.org/morpheus/, accessed on 11 September 2026). Hierarchical clustering was executed employing Spearman correlation as the distance metric combined with the complete linkage method, enabling two-way clustering of both triplicate injections and individual metabolite features to visualize distinct phytochemical accumulation patterns.

#### 4.4.2. Principal Component Analysis (PCA)

Prior to multivariate data analysis, the untargeted LC-MS peak area dataset comprising 162 secondary metabolites across all injection triplicates was subjected to logarithmic transformation (log_1*p*_) to stabilize variance and ensure a normal-like distribution of feature intensities. The transformed dataset was subsequently autoscaled (mean-centered and scaled to unit variance) to give equal weight to all variables regardless of their absolute abundance.

An unsupervised Principal Component Analysis (PCA) was performed to evaluate the intrinsic data structure, identify potential clustering tendencies, detect outliers, and visualize group separation among the investigated plant species (*Achillea*, *Calendula*, *Hypericum*, *Plantago*, and *Verbena*). All chemometric calculations, data preprocessing, and score plot visualizations were executed in Python (version 3.14), utilizing the scikit-learn library for PCA modeling, alongside pandas and numpy for data manipulation and matplotlib/seaborn for graphical representation.

## 5. Conclusions

The analytical framework utilizing UHPLC-ESI-HRMS/MS provided superior resolution and a comprehensive characterization of the phytochemical profiles of five established medicinal plants: *Verbena officinalis*, *Plantago major*, *Hypericum perforatum*, *Achillea millefolium*, and *Calendula officinalis*. The findings underscore the chemical diversity of the investigated extracts, with phenolic compounds representing the predominant chemical class and providing a basis for further biological investigation based on the activities previously reported for individual constituents. A major contribution of this study was the identification of 115 unique compounds previously unreported within these specific botanical species. The species-level distribution of these novel constituents—comprising 52 in *P. major*, 44 in *C. officinalis*, 44 in *H. perforatum*, 36 in *A. millefolium*, and 34 in *V. officinalis*—significantly expands and redefines current phytochemotaxonomic databases. From a methodological standpoint, these insights reaffirm the indispensable role of high-resolution mass spectrometry (HRMS) as the gold standard for the non-targeted chemoprofiling of highly complex plant substrates. Furthermore, the distinct variations observed among the investigated chemical profiles highlight the complementary chemical diversity of the five species. These differences provide a rationale for future studies evaluating whether selected extracts or their combinations exhibit complementary or synergistic biological effects. The chemical profiles generated in this study can serve as a basis for subsequent biological and pharmacological evaluation of the individual extracts and their combinations, with particular emphasis on determining whether the identified chemical diversity translates into relevant biological effects and whether selected combinations may have potential applications in wound care.

## Data Availability

The raw data supporting the conclusions of this article will be made available by the authors upon request.

## Supplementary Materials

The following supporting information can be downloaded at https://www.mdpi.com/article/10.3390/plants15203164/s1. Figure S1: Hierarchical cluster analysis (HCA) heatmap illustrating the distribution and relative abundance of 162 untargeted secondary metabolites across the investigated plant species (*Achillea*, *Calendula*, *Hypericum*, *Plantago*, and *Verbena* in triplicates). The heatmap was generated using Morpheus software based on Spearman correlation distance and complete linkage. Rows represent individual metabolites, while columns represent individual replicates. Color intensity ranges from light green; Figure S2: Top loading bar charts illustrating the variables with the highest absolute contribution (loadings) along the first two principal components (PC1 and PC2) derived from the PCA model of 162 untargeted secondary metabolites across the investigated botanical species; Table S1: LC-MS data on metabolites identified in five plant species; Table S2: Peak areas of 162 metabolites identified in five plant species (in triplicates).

## Abbreviations

The following abbreviations are used in this manuscript:

Am	*Achillea millefolium*
Co	*Calendula officinalis*
EtOH	Ethanol
HCA	Hierarchical cluster analysis
Hp	*Hypericum perforatum*
LC-MS	Liquid chromatography–mass spectrometry
*m*/*z*	Mass-to-charge ratio
MS	Mass spectrometry
PC1	First principal component
PC2	Second principal component
Pm	*Plantago major*
ppm	Parts per million
PPAP	Polycyclic polyprenylated acylphloroglucinol
Rt	Retention time
UHPLC-ESI-HRMS/MS	Ultra-high-performance liquid chromatography coupled with electrospray ionization high-resolution tandem mass spectrometry
Vo	*Verbena officinalis*
