# Supplementary material for: Metabolomic Fingerprinting and UHPLC-ESI-HRMS/MS Profiling of Five Medicinal Plants Associated with Wound Care
Source: Plants (Basel). 2026 Oct 15;15(20):3164. doi: 10.3390/plants15203164 (PMC13708942; doi:10.3390/plants15203164)
Supplement: Supplementary file 1 [file plants-15-03164-s001.zip › plants-4596230-supplementary/Table S1.pdf]

Table S1.

No	Compound name	t_R min	Molecular formula [M–H]⁻	Calculated mass *m/z*	Exact mass *m/z*	Δ ppm	MS² Fragments, (% Base Peak)	Vo	Pm	Hp	Am	Co	MSI confidence levels#
							Hydroxybenzoic acid derivatives						
**1**	**Galloyl pentoside**	0.46	$C_{12}H_{13}O_9^-$	301.05651	301.05561	2.98	101.06533(11), 119.05391(11), 125.02444(45), 149.99666(38), 168.00720(47), **301.05615**(100)	*	/	*	*	*	level 3
**2**	**Dihydroxybenzoyl pentosyl-hexoside**	0.48	$C_{18}H_{23}O_{13}^-$	447.11442	447.11414	0.61	108.02167(41), 152.01152(57), 284.03287(9), 285.0405(12), **447.11304**(100)	/	/	*	*	/	level 3
**3**	**Syringoyl hexoside 1**	0.48	$C_{15}H_{19}O_{10}^-$	359.09837	359.09882	-1.26	123.00872(91), 138.03201(45), 153.05583(16), 166.99918(9), 182.02200(29), **197.04526**(100)	*	*	+	*	*	level 3
**4**	**Hydroxybenzoyl hexoside 1**	0.48	$C_{13}H_{15}O_8^-$	299.07724	299.07726	-0.05	**93.03457**(100), 137.02420(60)	*	*	*	*	*	level 3
**5**	**Gallic acid**	0.48	$C_7H_5O_5^-$	169.01425	169.01414	0.65	81.03418(6), 97.02914(9), **125.02396**(100), 169.01378(7)	+	+	+	*	/	level 1
**6**	**3,4-Dihydroxybenzoic acid**	0.49	$C_7H_5O_4^-$	153.01933	153.01921	0.80	108.02164(23), **109.02956**(100), 153.02023(4)	+	+	+	+	+	level 1
**7**	**Vanilloyl hexoside**	0.63	$C_{14}H_{17}O_9^-$	329.08781	329.08791	-0.31	**108.02166**(100), 123.04517(19), 152.01152(34), 167.03493(93)	+	*	+	+	*	level 3
**8**	**Dihydroxybenzoyl hexoside**	0.64	$C_{13}H_{15}O_9^-$	315.07216	315.07224	-0.27	**108.02169**(100), 109.02957(37), 152.01155(35), 153.01935(30), 315.07208(46)	+	*	+	+	*	level 3
**9**	**Dihydroxybenzoyl pentoside**	0.83	$C_{12}H_{13}O_8^-$	285.06159	285.06163	-0.15	**108.02165**(100), 109.02965(11), 152.01144(37), 153.01935(23), 285.06198(28)	+	*	*	+	*	level 3
**10**	**4-Hydroxybenzoic acid**	1.13	$C_7H_5O_3^-$	137.02442	137.02388	3.96	**93.03420**(100)	*	+	+	/	+	level 1
**11**	**Hydroxybenzoyl hexoside 2**	1.18	$C_{13}H_{15}O_8^-$	299.07724	299.07727	-0.08	**93.03426**(100), 137.02386(76)	/	/	/	*	/	level 3
**12**	**Ellagic acid**	5.21	$C_{14}H_5O_8^-$	300.99899	300.99760	4.62	145.02916(9), 283.99542(8), **300.99774**(100)	/	/	/	+	/	level 1
**13**	**Hydroxybenzoic acid**	5.40	$C_7H_5O_3^-$	137.02442	137.02441	0.07	**93.03461**(100), 137.02470(4)	+	+	/	+	*	level 2
**14**	**Gallic acid-hydroxybenzoyl hexoside**	6.27	$C_{20}H_{19}O_{12}^-$	451.08820	451.08786	0.75	125.02401(17), 137.02415(30), 168.00600(43), 313.05594(8), 331.06699(17), **451.08691**(100)	/	/	/	*	/	level 3
**15**	**Dihydroxybenzoic acid-benzoyl hexoside**	6.37	$C_{20}H_{19}O_{10}^-$	419.09837	419.09781	1.33	108.02164(23), 109.02947(97), 152.01155(16), 153.01932(46), **419.09793**(100)	*	/	/	*	/	level 3
**16**	**Dihydroxybenzoic acid-dihydroxybenzoyl hexoside**	6.49	$C_{20}H_{19}O_{11}^-$	435.09329	435.09272	1.29	108.02172(12), 109.02947(63), 137.02446(63), 152.01146(11), 153.01936(58), **435.09299**(100)	/	/	*	*	*	level 3
							Hydroxycinnamic acid derivatives						
**17**	**Caffeoyl hexosyl-hexoside**	0.49	$C_{21}H_{27}O_{14}^-$	503.14063	503.13906	3.12	113.02464(9), 135.04523(47), 161.02438(26), **179.03514**(100)	*	*	/	*	/	level 3
**18**	**Caffeoyl hexoside**	0.68	$C_{15}H_{17}O_9^-$	341.08781	341.08712	2.01	133.02867(33), **135.04544**(100), 161.02472(58),	*	*	+	+	*	level 3

							179.03532(96)						
**19**	**3-*O*-Caffeoylquinic acid**	0.75	$C_{16}H_{17}O_9^-$	353.08781	353.08679	2.87	134.03690(13), 135.04466(55), 179.03435(40), **191.05537**(100), 353.08572(10)	*	+	+	+	+	level 2
**20**	**Feruloyl hexoside**$^-$	0.90	$C_{17}H_{21}O_{11}^-$	401.10894	401.10844	1.24	134.03755(65), **193.05095**(100)	/	*	*	/	/	level 3
**21**	**3-*O*-*p*-Coumaroylquinic acid**	1.17	$C_{16}H_{17}O_8^-$	337.09289	337.09302	-0.39	119.05026(49), **163.04008**(100), 173.04562(5), 191.05614(18)	/	/	+	/	/	level 2
**22**	***p*-Coumaroyl hexoside**	1.87	$C_{15}H_{17}O_8^-$	325.09289	325.09169	3.68	**119.04979**(100), 145.02954(3), 163.03941(60)	/	*	/	/	/	level 3
**23**	**Caffeic acid**	2.32	$C_9H_7O_4^-$	179.03498	179.03419	4.40	**135.04465**(100)	/	/	/	+	+	level 1
**24**	***p*-Coumaric acid**	4.55	$C_9H_7O_3^-$	163.04007	163.03962	2.73	93.03418(11), **119.04977**(100)	/	+	+	+	+	level 1
**25**	**5-*O*-Feruloylquinic acid**	4.64	$C_{17}H_{19}O_9^-$	367.10346	367.10288	1.56	173.04712(11), **191.05612**(100)	/	/	/	+	/	level 2
**26**	**Ferulic acid**	5.10	$C_{10}H_9O_4^-$	193.05063	193.04976	4.52	133.02904(10), **134.03687**(100), 149.05991(6), 178.02643(32), 193.05074(3)	+	+	+	/	/	level 1
**27**	**Sinapic acid**	5.18	$C_{11}H_{11}O_5^-$	223.06120	223.06101	0.83	**93.03421**(100), 121.02906(54), 149.02386(42), 164.04726(76), 193.01335(40), 208.03687(75)	/	/	/	+	+	level 1
**28**	**Dicaffeoylquinic acid 1**	5.62	$C_{25}H_{23}O_{12}^-$	515.11950	515.11902	0.93	135.04466(43), 161.02385(6), 179.03429(46), 191.05533(98), **353.08575**(100), 515.11713(10)	+	*	+	+	+	level 3
**29**	**Dicaffeoylquinic acid 2**	5.85	$C_{25}H_{23}O_{12}^-$	515.11950	515.11892	1.13	135.04510(49), 173.04552(43), 179.03496(58), 191.05611(80), **353.08719**(100), 515.11932(23)	/	/	/	+	+	level 3
**30**	**Rosmarinic acid**	5.85	$C_{18}H_{15}O_8^-$	359.07724	359.07635	2.48	123.04558(12), 133.02963(30), 135.04524(19), **161.02483**(100), 179.03479(22), 197.04582(46)	+	+	+	+	+	level 1
**31**	**Caffeoyl-feruloylquinic acid**	6.06	$C_{26}H_{25}O_{12}^-$	529.13515	529.13488	0.51	134.03731(60), 173.04538(15), 191.05606(48), 193.05052(78), 353.08664(10), **367.10269**(100)	/	/	/	*	*	level 3
**32**	***p*-Coumaric acid-*p*-coumaroyl hexoside**	6.19	$C_{24}H_{23}O_{10}^-$	471.12967	471.12947	0.43	**119.05038**(100), 145.03001(53), 163.04036(37), 213.0616(18), 307.08121(25), 471.13049(53)	/	*	/	/	/	level 3
**33**	**Dicaffeoylquinic acid 3**	6.27	$C_{25}H_{23}O_{12}^-$	515.11950	515.11758	3.72	135.04521(52), 173.04579(49), 179.03502(56), 191.05617(64), **353.08740**(100), 515.1178(26)	/	/	/	/	*	level 3
**34**	**Nepetoidin B**	7.06	$C_{17}H_{13}O_6^-$	313.07176	313.07168	0.25	133.03006(41), **161.02463**(100)	*	*	/	*	/	level 2
							Phenylethanoid glycosides						
**35**	**Hellicoside**	4.59	$C_{29}H_{35}O_{17}^-$	655.18797	655.18782	0.23	135.04512(5), 161.02441(83), 179.03511(13), 475.14481(7), 637.17725(6), **655.18793**(100)	/	+	/	/	/	level 3
**36**	**Hydroxy-scroside E**	5.14	$C_{30}H_{37}O_{17}^-$	669.20362	669.20287	1.13	151.03992(18), 160.01680(20), 175.04068(53), 193.05128(26), 651.20056(59), **669.20184**(100)	/	*	/	/	/	level 3
**37**	**Oxoverbascoside**	5.23	$C_{29}H_{33}O_{16}^-$	637.17741	637.17703	0.59	161.02438(87), 179.03502(14), 315.10828(4), **477.14011**(100), 619.16675(4)	*	*	/	/	/	level 3
**38**	**Plantamajoside**	5.25	$C_{29}H_{35}O_{16}^-$	639.19306	639.19251	0.86	135.04466(8), 161.02374(84), 179.03458(3),	*	+	*	/	*	level 3

							315.10764(3), 477.15906(13), **639.19025**(100)						
**39**	**Verbascoside**	5.43	$C_{29}H_{35}O_{15}^-$	623.19814	623.19776	0.62	161.02373(72), 314.04187(18), 315.05017(5), 461.16388(10), **623.19135**(100)	+	+	/	/	/	level 3
**40**	**Betonyoside B**	5.59	$C_{30}H_{37}O_{16}^-$	653.20871	653.20808	0.96	160.01653(18), 161.02429(9), 175.03998(41), 193.05052(8), 477.16077(17), **653.20844**(100)	*	*	/	/	/	level 3
**41**	**Leucosceptoside A**	5.77	$C_{30}H_{37}O_{15}^-$	637.21379	637.21334	0.71	160.01665(21), 161.02451(9), 175.04008(47), 193.05095(7), 461.16644(14), **637.21417**(100)	+	+	/	/	/	level 3
**42**	**2′-Acetyl-verbascoside**	5.80	$C_{31}H_{37}O_{16}^-$	665.20871	665.20856	0.22	135.04504(32), 161.02434(64), 167.03540(7), 179.03493(85), 487.14694(16), **665.20892**(100)	+	*	/	/	/	level 3
**43**	**Hemiphroside A**	5.94	$C_{31}H_{39}O_{16}^-$	667.22436	667.22413	0.35	113.02436(8), 134.03725(10), 160.01654(25), 175.03996(72), 193.05054(17), **667.22369**(100)	*	*	*	/	/	level 3
**44**	**Lancetoside**	6.29	$C_{29}H_{35}O_{14}^-$	607.20323	607.20337	-0.22	101.02448(6), 113.02425(11), 119.03466(4), 133.02953(12), 135.04443(6), **161.02379**(100)	/	*	/	/	/	level 3
							Iridoid glycosides						
**45**	**Verbenalin**$^-$	4.16	$C_{18}H_{25}O_{12}^-$	433.13515	433.13436	1.83	**101.02439**(100), 119.03529(7), 121.06626(5), 179.05716(11), 193.05095(13), 225.07678(99)	+	/	/	/	/	level 3
**46**	**Dihydroverbenalin**$^-$	4.90	$C_{18}H_{27}O_{12}^-$	435.15080	435.14875	4.70	101.02412(42), 125.02441(9), 143.25572(3), 195.06635(6), **227.09171**(100)	+	*	/	/	/	level 3
**47**	**Deoxyloganic acid**	5.18	$C_{16}H_{23}O_9^-$	359.13476	359.13398	2.17	109.06591(15), 135.08165(51), 153.09209(41), **197.08195**(100), 359.13406(28)	*	*	*	*	*	level 3
							Flavonoid C-glycosides						
**48**	**Apigenin 6-*C*-hexoside-8-*C*-hexoside**	4.36	$C_{27}H_{29}O_{15}^-$	593.15119	593.15217	-1.65	353.06717(28), 383.07919(13), **593.15210**(100)	/	+	/	/	/	level 2
**49**	**Apigenin 6-*C*-hexoside-8-*C*-pentoside**	4.87	$C_{26}H_{27}O_{14}^-$	563.14063	563.14061	0.03	353.06696(24), 383.07651(10), **563.14014**(100)	/	/	/	+	*	level 2
**50**	**Apigenin 8-*C*-glucoside (Vitexin)**	5.26	$C_{21}H_{19}O_{10}^-$	431.09837	431.09764	1.71	283.06009(60), 311.05499(92), 323.05460(3), 341.06525(7), **431.09644**(100)	+	+	/	+	+	level 1
**51**	**Luteolin 8-*C*-pentoside**	5.53	$C_{20}H_{17}O_{10}^-$	417.08272	417.08216	1.33	133.02966(27), 284.03229(12), 299.05621(21), **327.05124**(100), 357.06128(23), 417.08200(73)	/	/	*	*	/	level 2
							Flavonoid O-glycosides						
**52**	**Quercetin 3,7-di-*O*-hexoside**	4.46	$C_{27}H_{29}O_{17}^-$	625.14102	625.13971	2.09	151.00374(11), 300.02805(37), 301.03433(70), **463.08853**(100), 625.13971(49)	/	/	*	/	/	level 2
**53**	**Luteolin 7-*O*-(2"-hexuronyl)-hexuronide**	4.67	$C_{27}H_{25}O_{18}^-$	637.10464	637.10454	0.16	113.02393(16), 175.02391(4), 193.03452(9), 285.03912(66), 351.05453(28), **637.10138**(100)	+	/	/	/	/	level 2
**54**	**Quercetin 3-*O*-hexuronide-7-O-hexoside**	4.78	$C_{27}H_{27}O_{18}^-$	639.12029	639.12177	-2.33	151.00415(21), **301.03561**(100), 302.03171(10), 463.09552(19), 639.12177(98)	*	/	*	*	/	level 2

**55**	**Myricetin 3-*O*-hexoside**	4.85	$C_{21}H_{19}O_{13}^-$	479.08318	479.08313	0.10	151.00417(7), 271.02383(27), 287.02032(20), 316.02252(93), 317.02838(9), **479.08313**(100)	*	+	+	/	/	level 2
**56**	**Quercetin 3-*O*-(2'',6''-dirhamnosyl)-hexoside**	4.93	$C_{33}H_{39}O_{20}^-$	755.20402	755.20371	0.40	178.99879(2), 271.02454(5), 300.02759(61), 301.03470(5), **755.20361**(100)	*	/	/	/	+	level 2
**57**	**Quercetin 3-*O*-pentoside-7-*O*-hexoside**	5.01	$C_{26}H_{27}O_{16}^-$	595.13046	595.12993	0.89	**300.02631**(100), 301.03403(36), 433.07587(18), 463.08615(22), 595.12677(90)	/	/	*	+	*	level 2
**58**	**Isorhamnetin 3-*O*-(6''-hexosyl)-hexoside-7-*O*-hexoside**	5.02	$C_{34}H_{41}O_{21}^-$	785.21458	785.21290	2.15	299.01935(7), 314.04373(27), 315.05219(20), 623.16107(12), **785.21289**(100)	/	/	/	/	*	level 2
**59**	**Apigenin 7-*O*-(2''-hexuronyl)-hexuronide**	5.07	$C_{27}H_{25}O_{17}^-$	621.10972	621.10971	0.02	113.02439(31), 175.02470(8), 193.03526(16), 269.04559(27), **351.05603**(100)	+	*	/	/	/	level 2
**60**	**Isorhamnetin 3-*O*-(6''-hexosyl)-hexoside**	5.17	$C_{28}H_{31}O_{17}^-$	639.15667	639.15587	1.26	179.03682(15), 299.02206(27), 314.04642(42), 315.05319(14), 639.16174(100)	/	/	/	/	*	level 2
**61**	**Quercetin 3-*O*-(2''-pentosyl)-hexuronide**	5.20	$C_{26}H_{25}O_{17}^-$	609.10976	609.10908	1.12	151.00366(25), 178.99860(13), 300.02771(6), **301.03534**(100), 609.1098(90)	/	/	/	+	/	level 2
**62**	**Isorhamnetin 3-*O*-(2'',6''-dirhamnosyl)-hexoside**	5.21	$C_{34}H_{41}O_{20}^-$	769.21967	769.21939	0.37	299.01855(10), 314.0419(31), 315.04919(6), **769.21606**(100)	/	/	/	/	+	level 2
**63**	**Quercetin 3-*O*-(6''-rhamnosyl)-glucoside (Rutin)**	5.21	$C_{27}H_{29}O_{16}^-$	609.14611	609.14473	2.27	151.00308(4), 178.99734(2), 271.02377(16), 300.02637(61), 301.03400(24), **609.14325**(100)	+	/	+	+	+	level 1
**64**	**Isorhamnetin 3-*O*-(2''-pentosyl-6''-rhamnosyl)-hexoside**	5.23	$C_{33}H_{39}O_{20}^-$	755.20402	755.20325	1.02	271.02579(4), 299.01987(14), 300.02542(3), 314.04297(39), 315.05121(10), **755.20325**(100)	/	/	/	/	*	level 2
**65**	**Kaempferol 3-*O*-pentoside-7-*O*-hexoside**	5.26	$C_{26}H_{27}O_{15}^-$	579.13554	579.13587	-0.56	152.01154(12), 255.03128(17), 284.03357(40), 417.10254(27), **579.13544**(100)	*	/	*	*	*	level 2
**66**	**Quercetin 3-*O*-galactoside (Hyperoside)**	5.30	$C_{21}H_{19}O_{12}^-$	463.08820	463.08738	1.78	151.00348(10), 243.02998(10), 271.02466(37), 300.02750(61), 301.03531(41), **463.08737**(100)	*	+	+	/	+	level 1
**67**	**Luteolin 7-*O*-hexuronide**	5.30	$C_{21}H_{17}O_{12}^-$	461.07255	461.07187	1.48	**285.04047**(100), 461.07220(9)	+	+	*	+	*	level 2
**68**	**Procyanidin dimer B type**	5.31	$C_{30}H_{25}O_{12}^-$	577.13515	577.13382	2.30	**125.02445**(100), 245.08221(14), 289.07184(64), 407.07687(40), 425.08737(48), 577.13513(67)	/	/	+	/	/	level 2
**69**	**Taxifolin 3-*O*-rhamnoside**	5.31	$C_{21}H_{21}O_{11}^-$	449.10894	449.10828	1.45	107.0139(21), 125.02449(23), **151.00374**(100), 178.99872(9), 285.04059(51), 303.05112(31)	*	*	+	*	/	level 2
**70**	**Kaempferol 3-*O*-rhamoside-7-*O*-rhamnoside**	5.37	$C_{27}H_{29}O_{14}^-$	577.15628	577.15424	3.54	255.03082(18), 283.02484(69), 285.04065(79), 430.08853(14), 431.0994(27), **577.15515**(100)	/	*	/	/	/	level 2
**71**	**Luteolin 7-*O*-glucoside (Cynaroside)**	5.38	$C_{21}H_{19}O_{11}^-$	447.09329	447.09245	1.87	107.01395(6), 133.02927(8), 151.00377(9), 284.03256(60), 285.04031(74), **447.09280**(100)	+	+	/	+	/	level 1
**72**	**Patuletin 3-*O*-hexuronide**	5.39	$C_{22}H_{19}O_{14}^-$	507.07803	507.07779	0.46	165.99126(13), 287.01947(48), 288.02829(8), 316.02286(73), **331.04581**(100)	/	/	/	*	/	level 2
**73**	**Patuletin 3-*O*-hexoside**	5.43	$C_{22}H_{21}O_{13}^-$	493.09877	493.09815	1.25	165.99075(8), 315.0148(21), 316.02274(19), 330.03809(14), 331.04510(48), **493.09705**(100)	/	*	/	*	+	level 2

**74**	**Kaempferol 3-*O*-(6"-rhamnosyl)-hexoside**	5.48	$C_{27}H_{29}O_{15}^{-}$	593.15119	593.15150	-0.51	227.03488(10), 229.05057(5), 255.02998(21), 284.03271(48), 285.04047(56), **593.15149**(100)	*	/	+	+	+	level 2
**75**	**Quercetin 3-*O*-(6"-acetyl)-hexoside**	5.48	$C_{23}H_{21}O_{13}^{-}$	505.09877	505.09822	1.08	243.03012(10), 255.02994(21), 271.02481(52), **300.02753**(100), 301.03464(8), 505.09857(91)	/	/	+	+	+	level 2
**76**	**Quercetin 3-*O*-(6"-malonyl)-hexoside**	5.49	$C_{24}H_{21}O_{15}^{-}$	549.08859	549.08817	0.78	151.00378(5), 178.99841(3), 271.02478(39), 300.02747(91), 301.03497(16), **505.09848**(100)	/	*	*	+	+	level 2
**77**	**Quercetin 3-*O*-pentoside**	5.49	$C_{20}H_{17}O_{11}^{-}$	433.07764	433.07700	1.46	243.02991(15), 255.03014(26), 271.02484(61), 300.02759(95), 301.03546(19), **433.07721**(100)	/	*	+	+	*	level 2
**78**	**Isorhamnetin 3-*O*-hexuronide**	5.49	$C_{22}H_{19}O_{13}^{-}$	491.08311	491.08272	0.81	113.02403(10), 243.02879(7), 255.02884(10), 271.02371(23), 300.02643(39), **315.04974**(100)	+	*	/	*	/	level 2
**79**	**Isorhamnetin 3-*O*-(2"-pentosyl)-hexuronide**	5.51	$C_{27}H_{27}O_{17}^{-}$	623.12546	623.12341	3.28	255.03055(5), 271.0238(7), 300.02725(43), 307.06732(8), **315.05084**(100), 623.12341(54)	*	*	/	*	*	level 2
**80**	**Isorhamnetin 3-*O*-(6"-rhamnosyl)-glucoside**	5.55	$C_{28}H_{31}O_{16}^{-}$	623.16176	623.16155	0.34	299.01962(24), 300.02756(19), 314.04321(29), 315.05096(62), **623.16150**(100)	+	+	+	+	+	level 1
**81**	**Quercetin 3-*O*-rhamnoside (Quercitrin)**	5.62	$C_{21}H_{19}O_{11}^{-}$	447.09329	447.09312	0.37	151.00347(12), 178.99861(7), 271.024817(43), 300.02726(70), 301.03534(48), **447.09311**(100)	/	/	+	+	*	level 1
**82**	**Isorhamnetin 3-*O*-(2"-acetyl-6"-rhamnosyl)-hexoside**	5.65	$C_{30}H_{33}O_{17}^{-}$	665.17232	665.17215	0.25	285.04028(5), 299.01978(36), 300.02628(3), 314.04327(66), 315.05038(9), **665.17212**(100)	*	*	/	/	*	level 2
**83**	**Apigenin 7-*O*-(2"-pentosyl)-hexoside**	5.67	$C_{26}H_{27}O_{14}^{-}$	563.14063	563.14016	0.83	**269.04544**(100)	*	*	/	+	/	level 2
**84**	**Quercetin 3-*O*-hexuronide**	5.68	$C_{21}H_{17}O_{13}^{-}$	477.06746	477.06563	3.85	107.01399(6), 109.02945(7), 121.02943(6), 151.00365(28), 178.99846(11), **301.03525**(100)	/	+	+	+	+	level 2
**85**	**Isorhamnetin 3-*O*-glucoside**	5.68	$C_{22}H_{21}O_{12}^{-}$	477.10385	477.10127	5.41	271.02368(27), 299.01859(6), 301.03421(10), 314.04190(41), 315.04901(6), **477.10126**(100)	/	*	+	/	+	level 1
**86**	**Kaempferol 3-*O*-glucoside (Astragalin)**	5.69	$C_{21}H_{19}O_{11}^{-}$	447.09329	447.09086	5.43	151.00383(5), 227.03485(25), 255.02982(27), 284.03250(50), 285.04037(54), **447.09286**(100)	/	/	/	/	+	level 1
**87**	**Apigenin 7-*O*-hexuronide**	5.72	$C_{21}H_{17}O_{11}^{-}$	445.07764	445.07696	1.53	113.02461(21), 117.03465(8), 175.02496(9), **269.04523**(100), 445.07764(6)	+	*	/	+	/	level 2
**88**	**Chrysoeriol 7-*O*-(6"-rhamnosyl)-hexoside**	5.73	$C_{28}H_{31}O_{15}^{-}$	607.16684	607.16692	-0.12	135.04755(8), 145.02956(25), 161.02495(10), 284.03244(45), **299.05606**(100), 607.18274(60)	*	/	*	+	/	level 2
**89**	**Quercetin 7-*O*-hexoside**	5.73	$C_{21}H_{19}O_{12}^{-}$	463.08820	463.08769	1.10	151.00369(47), 178.99855(18), 271.02527(9), 300.02774(22), **301.03540**(100), 463.08792(63)	*	*	+	*	+	level 2
**90**	**Chrysoeriol 7-*O*-hexoside**	5.75	$C_{22}H_{21}O_{11}^{-}$	461.10894	461.10886	0.16	284.03229(7), 285.04050(34), 298.04807(11), 299.05643(6), 446.08521(14), **461.10886**(100)	/	*	/	+	+	level 2
**91**	**Isorhamnetin 3-*O*-pentoside-7-*O*-pentoside**	5.76	$C_{26}H_{27}O_{15}^{-}$	579.13554	579.13593	-0.67	271.02539(14), 299.02026(29), 314.04370(50), **579.13593**(100)	/	/	/	/	*	level 2
**92**	**Kaempferol 3-*O*-(6"-malonyl)-**	5.76	$C_{24}H_{21}O_{14}^{-}$	533.09368	533.09342	0.48	173.04449(25), 227.03435(9), 255.03038(25),	/	*	*	*	*	level 2

	**hexoside**						284.03119(67), 285.03961(56), **489.10098**(100)						
**93**	**Isorhamnetin 3-*O*-(2"-caffeoyl-6"-pentosyl)-hexoside**	5.77	$C_{36}H_{35}O_{19}^{-}$	771.17780	771.17902	-1.58	314.04474(18), 315.05029(13), 609.14215(18), **771.17902**(100)	/	/	/	/	*	level 2
**94**	**Quercetin 3-*O*-(2"-acetyl)-rhamnoside**	5.79	$C_{23}H_{21}O_{12}^{-}$	489.10385	489.10341	0.90	227.03516(27), 255.0309(46), **284.03238**(100), 285.04208(21), 489.10468(70)	/	*	*	*	*	level 2
**95**	**Hispidulin 7-*O*-hexuronide**	5.83	$C_{22}H_{19}O_{12}^{-}$	475.08820	475.08793	0.58	113.02438(29), 165.99081(6), 175.02481(10), 283.02554(4), **284.03268**(100), 299.05615(97)	/	*	*	/	*	level 2
**96**	**Isorhamnetin 3-*O*-(6"-malonyl)-hexoside**	5.87	$C_{25}H_{23}O_{15}^{-}$	563.10423	563.10413	0.17	271.0249(23), 299.01978(60), 300.02768(21), 314.04346(50), 315.05112(64), **519.11438**(100)	*	*	/	*	+	level 2
**97**	**Isorhamnetin 3-*O*-(6"-acetyl)-hexoside**	5.88	$C_{24}H_{23}O_{13}^{-}$	519.11442	519.11392	0.95	271.02475(38), 299.01978(61), 300.0275(20), 314.04333(53), 315.05109(61), **519.11377**(100)	/	*	/	+	+	level 2
**98**	**Tricin 7-*O*-hexoside**	5.91	$C_{23}H_{23}O_{12}^{-}$	491.11950	491.11906	0.90	271.02484(15), 285.03894(12), 313.03497(26), 461.07059(19), 476.09604(11), **491.11823**(100)	/	+	*	*	*	level 2
**99**	**Quercetin 7-*O*-pentoside**	5.98	$C_{20}H_{17}O_{11}^{-}$	433.07764	433.07719	1.03	107.01383(11), 121.02927(16), 151.00316(59), 178.99841(20), 300.02725(21), **301.03448**(100)	/	/	*	/	/	level 2
**100**	**Apigenin 7-*O*-(6"-acetyl)-hexoside**	6.00	$C_{23}H_{21}O_{11}^{-}$	473.10894	473.10832	1.31	256.04028(13), 268.03741(74), 269.04669(35), **473.10831**(100)	/	*	/	+	+	level 2
**101**	**Apigenin 7-*O*-glucoside (Apigetrin)**	6.03	$C_{21}H_{19}O_{10}^{-}$	431.09837	431.09782	1.29	107.01402(4), 151.00349(5), 211.04028(4), 268.03760(95), 269.04526(20), **431.09781**(100)	/	/	+	/	/	level 1
**102**	**Luteolin 7-*O*-(6"-hexuronyl)-hexoside**	6.06	$C_{27}H_{27}O_{17}^{-}$	623.12546	623.12734	-3.02	113.02651(6), **285.04044**(100), 623.12933(35)	/	*	/	/	/	level 2
**103**	**Kaempferol 3-*O*-(6"-acetyl)-hexoside**	6.06	$C_{23}H_{21}O_{12}^{-}$	489.10385	489.10357	0.58	243.02997(11), 255.02992(22), 271.02481(52), 300.02759(89), 301.03510(11), **489.10336**(100)	/	*	*	/	/	level 2
**104**	**Apigenin 7-*O*-(6"-caffeoyl)-hexoside**	6.45	$C_{30}H_{25}O_{13}^{-}$	593.13007	593.13129	-2.07	161.02501(33), 213.00903(8), 269.04605(87), **593.13129**(100)	*	*	/	/	/	level 2
**105**	**Eriodictyol-3',8′′-quercetin**	6.47	$C_{30}H_{19}O_{13}^{-}$	587.08311	587.08080	3.94	133.02946(3), 151.00374(6), **285.03949**(100), 301.03525(10)	/	/	/	*	/	level 2
**106**	**Isosakuranetin 7-*O*-(6"-rhamnosyl)-hexoside**	6.61	$C_{28}H_{33}O_{14}^{-}$	593.18758	593.18531	3.82	151.00238(7), 164.01111(10), 241.08551(6), 270.05282(20), 285.07590(98), **593.18530**(100)	*	/	/	/	/	level 2
**107**	**3′,8′′-Biapigenin**	7.11	$C_{30}H_{17}O_{10}^{-}$	537.08272	537.08182	1.67	151.00362(42), 267.02975(5), 385.07108(35), 443.04004(12), **537.08240**(100)	*	+	+	*	*	level 2
							Flavonoid aglycones						
**108**	**Catechin**	3.85	$C_{15}H_{13}O_{6}^{-}$	289.07176	289.07064	3.87	137.02402(27), 151.03934(21), 179.03409(23), 203.07068(35), 205.04967(29), **289.07065**(100)	/	/	+	/	/	level 1
**109**	**Taxifolin**	5.24	$C_{15}H_{11}O_{7}^{-}$	303.05103	303.04982	3.99	**125.02398**(100), 151.00314(10), 175.03958(13), 177.01877(15), 217.04985(10), 285.03922(57)	/	+	*	+	*	level 1

**110**	**Myricetin**	5.94	$C_{15}H_9O_8^-$	317.03029	317.02909	3.79	107.01352(14), 109.02908(30), 137.02391(30), 151.00314(35), 178.99791(23), **317.02905**(100)	/	/	+	/	/	level 1
**111**	**Patuletin**	5.97	$C_{16}H_{11}O_8^-$	331.04594	331.04475	3.60	165.99086(34), 316.02261(95), **331.04742**(100)	/	/	*	*	/	level 2
**112**	**Hispidulin**	6.11	$C_{16}H_{11}O_6^-$	299.05611	299.05583	0.95	**284.03159**(100), 299.05438(37)	/	+	*	/	/	level 1
**113**	**Eriodictyol**	6.41	$C_{15}H_{11}O_6^-$	287.05611	287.05479	4.60	107.01360(11), **135.04468**(100), 151.00314(73), 287.04749(9)	/	/	/	+	*	level 1
**114**	**Luteolin**	6.44	$C_{15}H_9O_6^-$	285.04046	285.04050	-0.13	107.01349(5), 133.02904(52), 149.02417(3), 151.0031(5), 175.03914(3), **285.03937**(100)	+	+	+	+	+	level 1
**115**	**Quercetin**	6.46	$C_{15}H_9O_7^-$	301.03538	301.03413	4.15	107.01384(20), 121.0295(21), 151.00368(55), 178.99854(27), 273.03958(4), **301.03534**(100)	+	+	+	+	+	level 1
**116**	**Axillarin**	6.66	$C_{17}H_{13}O_8^-$	345.06159	345.06092	1.94	165.99077(8), 287.0199(26), 315.01495(28), **330.03790**(100), 345.06125(44)	/	/	/	*	*	level 2
**117**	**Apigenin**	6.85	$C_{15}H_9O_5^-$	269.04555	269.04459	3.54	107.01340(6), 117.03416(54), 149.02415(4), 151.00316(4), **269.04453**(100)	+	+	+	+	+	level 1
**118**	**Naringenin**	6.87	$C_{15}H_{11}O_5^-$	271.06120	271.06117	0.11	119.04977(81), **151.00400**(100), 271.05926(39)	+	+	+	+	*	level 1
**119**	**Chrysoeriol**	6.94	$C_{16}H_{11}O_6^-$	299.05611	299.05611	0.02	**284.03156**(100), 299.05499(36)	+	*	*	+	*	level 1
**120**	**Kaempferol**	6.96	$C_{15}H_9O_6^-$	285.04046	285.04053	-0.25	117.03484(4), 143.05022(3), 151.00446(3), 185.06065(2), 211.04059(2), **285.04053**(100)	+	*	+	+	+	level 1
**121**	**Hesperetin**	6.97	$C_{16}H_{13}O_6^-$	301.07180	301.07095	2.81	151.00322(24), 164.01097(22), 242.05750(9), 286.04660(20), **301.07095**(100)	/	*	/	*	/	level 1
**122**	**6-Hydroxykaempferol 3,6-dimethyl ether**	7.03	$C_{17}H_{13}O_7^-$	329.06668	329.06659	0.26	314.04117(5), **329.07043**(100)	*	*	/	*	*	level 2
**123**	**Isorhamnetin**	7.04	$C_{16}H_{11}O_7^-$	315.05103	315.05102	0.02	107.01341(11), 151.00307(13), 255.02898(3), 271.02362(4), 300.02640(36), **315.04990**(100)	/	+	+	+	+	level 1
**124**	**Centaureidin**	7.25	$C_{18}H_{15}O_8^-$	359.07724	359.07658	1.84	258.01660(27), 286.01193(50), 301.03543(23), 314.00699(9), 329.03064(25), **344.05304**(100)	/	/	/	+	*	level 2
**125**	**Pectolinarigenin**	7.49	$C_{17}H_{13}O_6^-$	313.07176	313.07154	0.70	117.03403(33), 163.00244(27), 255.02902(36), 283.02600(40), 298.04788(67), **313.07123**(100)	/	*	/	/	/	level 2
**126**	**Chrysin**	7.94	$C_{15}H_9O_4^-$	253.05063	253.05063	0.01	65.00305(18), 107.01316(3), 119.04978(4), 143.04973(7), 145.02927(3), **253.04974**(100)	/	*	/	+	/	level 1
**127**	**Acacetin**	7.99	$C_{16}H_{11}O_5^-$	283.06120	283.06108	0.40	63.02386(13), 74.27455(10), 211.03963(16), 268.03699(74), **283.05991**(100)	+	*	+	+	*	level 1
**128**	**Pinocembrin**	8.02	$C_{15}H_{11}O_4^-$	255.06628	255.06514	4.48	107.01347(9), 145.06549(6), 151.00342(7), 171.04485(7), 213.05446(7), **255.06528**(100)	/	*	*	*	/	level 1
							Xanthone glycosides						
**129**	**Trihydroxyxanthone *C*-hexoside**	4.87	$C_{19}H_{17}O_{10}^-$	405.08272	405.08217	1.37	257.04712(40), **285.04102**(100), 405.08215(92)	/	/	*	/	/	level 3

**130**	**Trihydroxyxanthone *O*-hexoside**	6.22	$C_{19}H_{17}O_{10}^-$	405.08272	405.08226	1.13	187.04137(11), **243.03003**(100)	/	/	*	/	/	level 3
**131**	**Mangiferin**	4.99	$C_{19}H_{17}O_{11}^-$	421.07764	421.07689	1.78	258.0173(14), 271.02374(19), 273.04001(33), **301.03436**(100), 331.04559(16), 421.07571(13)	/	/	+	/	/	level 1
							Xanthone aglycones						
**132**	**Trihydroxy-methoxyxanthone**	6.93	$C_{14}H_9O_6^-$	273.04046	273.04106	-2.18	185.02441(6), 202.02716(18), 258.01697(73), **273.04059**(100)	/	/	*	/	/	level 3
**133**	**Dihydroxy-methoxyxanthone 1**	7.41	$C_{14}H_9O_5^-$	257.04555	257.04559	-0.15	103.01850(28), 158.03610(13), 187.00269(32), **242.02127**(100), 257.04419(35)	/	/	*	/	/	level 3
**134**	**Dihydroxy-dimethoxyxanthone**	7.89	$C_{15}H_{11}O_6^-$	287.05611	287.05612	-0.02	145.02957(16), 201.0193(79), 229.01483(18), 257.00903(27), **272.03262**(100), 287.05621(23)	/	/	*	/	/	level 3
**135**	**Dihydroxy-methoxyxanthone 2**	8.03	$C_{14}H_9O_5^-$	257.04555	257.04556	-0.07	158.03670(14), 172.01555(8), 186.03137(11), 214.02626(26), **242.02098**(100), 257.0444(97)	/	/	*	/	/	level 3
**136**	**Ugaxanthone**	9.40	$C_{18}H_{15}O_6^-$	327.08741	327.08734	0.23	243.02870(7), 271.02411(12), 283.02371(32), 297.03796(5), **327.08621**(100)	/	/	*	/	/	level 2
							Naphthodianthrones						
**137**	**Protopseudohypericin**	10.04	$C_{30}H_{17}O_9^-$	521.08781	521.08731	0.95	423.08771(5), 477.09799(3), 479.07803(2), **521.08734**(100)	/	/	+	/	/	level 2
**138**	**Hypericin**	11.63	$C_{30}H_{15}O_8^-$	503.07724	503.07668	1.12	405.07611(3), 433.07208(3), **503.07697**(100)	/	/	+	/	/	level 2
							Acylphloroglucinols						
**139**	**Hyperforatone I**	10.80	$C_{35}H_{53}O_6^-$	569.38476	569.38431	0.79	271.09778(8), 313.14450(15), 383.22229(40), 429.26437(5), 431.24445(4), **569.38470**(100)	/	/	+	/	/	level 2
**140**	**Norhyperpalum E**	11.25	$C_{35}H_{53}O_5^-$	553.38989	553.38951	0.68	271.09796(9), 313.14447(14), 383.22232(34), 413.26874(5), 415.24719(4), **553.38739**(100)	/	/	*	/	/	level 2
**141**	**Hyperwilsone F**	11.61	$C_{36}H_{53}O_6^-$	581.38476	581.38439	0.64	275.1272(7), 345.20514(23), 397.24042(13), 549.3562(33), **581.38342**(100)	/	/	*	/	/	level 2
**142**	**Hookerione B**	11.74	$C_{35}H_{49}O_5^-$	549.35855	549.35818	0.67	313.14294(20), 315.15869(8), 327.15872(8), 383.22067(15), 397.19574(14), **549.35583**(100)	/	/	*	/	/	level 2
**143**	**Furohyperforin A**	11.74	$C_{32}H_{45}O_5^-$	509.32725	509.32679	0.89	287.09247(10), 289.10800(6), 357.17029(11), **509.32767**(100)	/	/	+	/	/	level 2
**144**	**32-*epi*-Hyperforatin E**	11.77	$C_{35}H_{51}O_6^-$	567.36911	567.36880	0.56	275.12823(9), 313.14481(6), 355.22607(8), 357.24243(10), 535.34247(23), 567.36902(100)	/	/	+	/	/	level 2
**145**	**Hyperforatone J**	11.93	$C_{35}H_{53}O_6^-$	569.38476	569.38419	1.00	277.10828(8), 347.14871(18), 347.18823(9), 349.16489(12), 417.22787(27), **569.38458**(100)	/	/	+	/	/	level 2
**146**	**Hyperpatuone M**	12.04	$C_{36}H_{55}O_6^-$	583.40041	583.39998	0.74	347.18607(13), 361.16513(18), 363.18091(11), 431.24408(25), 487.30667(15), **583.39990**(100)	/	/	*	/	/	level 2

**147**	**Hyperuralone F**	12.13	$C_{36}H_{53}O_7^-$	597.37968	597.37942	0.43	313.14706(6), 375.21918(11), 383.22125(12), 445.26251(18), **597.41327**(100)	/	/	*	/	/	level 2
**148**	**Hyperscabin F**	12.27	$C_{34}H_{49}O_4^-$	521.36363	521.36329	0.66	299.12943(14), 301.14386(17), 369.20651(24), 453.30045(20), 521.08136(36), **521.36334**(100)	/	/	*	/	/	level 2
**149**	**Hyperfirin**	12.39	$C_{30}H_{43}O_4^-$	467.31668	467.31602	1.43	219.06555(11), 287.1279(36), 329.17480(32), **467.31467**(100)	/	/	+	/	/	level 2
**150**	**Furohyperforin**	12.42	$C_{35}H_{51}O_5^-$	551.37420	551.37368	0.94	259.09799(19), 275.12842(10), 329.17920(9), 343.19058(17), 411.25330(22), **551.37378**(100)	/	/	+	/	/	level 2
**151**	**Hyperforin**	12.54	$C_{35}H_{51}O_4^-$	535.37928	535.37873	1.03	313.14401(11), 313.18225(7), 315.15982(10), 383.22235(26), 398.24646(7), **535.37878**(100)	/	/	+	/	/	level 2
**152**	**Furoadhyperforin**	12.62	$C_{36}H_{53}O_5^-$	565.38985	565.38893	1.63	273.11322(15), 275.12888(4), 343.19183(7), 413.23297(6), 425.26929(29), **565.38947**(100)	/	/	+	/	/	level 2
**153**	**Hyperscabin C**	12.62	$C_{35}H_{53}O_5^-$	553.38985	553.38834	2.72	261.11331(12), 331.15479(16), 331.19409(9), 333.17078(13), 401.23276(23), **553.38965**(100)	/	/	*	/	/	level 2
**154**	**Adhyperfirin**	12.69	$C_{31}H_{45}O_4^-$	481.33233	481.33167	1.39	233.08147(11), 289.14578(5), 301.14420(34), 343.19058(31), **481.33212**(100)	/	/	+	/	/	level 2
**155**	**Adhyperforin**	12.74	$C_{36}H_{53}O_4^-$	549.39493	549.39459	0.63	285.11337(11), 313.18103(9), 327.16025(11), 329.17581(8), 397.23776(23), **549.39496**(100)	/	/	+	/	/	level 2
							Other metabolites						
**156**	**Aesculin**	0.48	$C_{15}H_{15}O_9^-$	339.07216	339.07192	0.69	105.03421(8), 133.02899(12), **177.01859**(100)	/	*	/	*	*	level 1
**157**	**Quinic acid**	0.48	$C_7H_{11}O_6^-$	191.05610	191.05603	0.34	85.02946(46), 93.03456(17), 111.04517(4), 127.04012(8), 173.04558(3), **191.05603**(100)	+	+	+	+	+	level 1
**158**	**Tuberonic acid hexoside**	4.29	$C_{18}H_{27}O_9^-$	387.16606	387.16542	1.64	101.02404(20), 113.02412(4), 119.03455(4), 207.10190(9), 225.07605(5), **387.16437**(100)	*	*	*	*	*	level 3
**159**	**Fraxidin**	5.17	$C_{11}H_9O_5^-$	221.04555	221.04544	0.50	107.01387(23), 135.00885(27), 163.00352(22), 190.9985(86), **206.02202**(100), 221.04517(6)	/	/	/	+	*	level 2
**160**	**Benzoyl-malic acid**	5.23	$C_{11}H_9O_6^-$	237.04046	237.04046	0.03	**121.02963**(100)	+	/	/	/	*	level 3
**161**	**5,7-Dihydroxy-2-(2-methylpropyl)-4H-1-benzopyran-4-one**	5.55	$C_{13}H_{13}O_4^-$	233.08193	233.08183	0.43	**189.05612**(100), 233.08266(49)	/	/	*	/	*	level 2
**162**	**Phlorizin**	6.00	$C_{21}H_{23}O_{10}^-$	435.12967	435.12736	5.31	119.04980(7), 123.04471(20), 125.02394(11), 167.03433(52), 179.03432(6), **273.07578**(100)	*	/	+	*	/	level 1

Vo – *Verbena officinalis*; Pm – *Plantago major*; Hp – *Hypericum perforatum*; Am – *Achillea millefolium*; Co – *Calendula officinalis*; Δ – mass accuracy; ~ - HCOOH adduct; / - not identified in the present study; + - identified in the present study and previously reported in the available literature; * - identified in the present study and previously not reported in the available literature; [#]MSI confidence levels: Level 1: Identified metabolite (Requires a full match against an authentic chemical standard analyzed in your laboratory under the exact same analytical conditions); Level 2: Putatively Annotated Compound (Based on a high-quality match to a public/commercial spectral libraries without an in-house authentic standard; typically matches accurate parent mass and fragment ions); Level 3: Putatively characterized class (The precise chemical structure remains uncertain, but spectral evidence (such as characteristic fragment ions, adduct patterns, or neutral losses) confidently places the feature within a specific compound class).
